# Exploring the Potential of Plant Bioactive Compounds against Male Infertility: An In Silico and In Vivo Study

**DOI:** 10.3390/molecules28237693

**Published:** 2023-11-21

**Authors:** Muhammad Jahangeer, Ghulam Mustafa, Naveed Munir, Sibtain Ahmed, Khalid Mashai Al-Anazi

**Affiliations:** 1 Department of Biochemistry, Government College University Faisalabad, Faisalabad 38000, Pakistan; 2 School of Health Sciences, Department of Biomedical Laboratory Sciences, University of Management and Technology, Lahore 54782, Pakistan; naveed.munir@umt.edu.pk; 3 Scripps Institution of Oceanography, University of California San Diego, 9500 Gilman Drive, La Jolla, CA 92093, USA; 4 Department of Biochemistry, Bahauddin Zakariya University, Multan 60800, Pakistan; 5 Department of Zoology, College of Science, King Saud University, Riyadh 11451, Saudi Arabia; kalanzi@ksu.edu.sa

**Keywords:** ADAM17, druggability, molecular docking, molecular dynamics simulation, phytochemicals, spermatogenesis, nicotine-induced male infertility

## Abstract

Infertility is a well-recognized multifactorial problem affecting the majority of people who struggle with infertility issues. In recent times, among infertility cases, the male factor has acquired importance, and now it contributes to approximately half of the infertility cases because of different abnormalities. In the current study, we used natural phytochemicals as potential drug-lead compounds to target different receptor proteins that are involved in the onset of male infertility. A set of 210 plant phytochemicals were docked counter to active site residues of sex hormone-binding globulin, a disintegrin and metalloproteinase 17, and DNase I as receptor proteins. On the basis of binding scores and molecular dynamics simulation, the phytochemicals tricin, quercetin, malvidin, rhamnetin, isorhamnetin, gallic acid, kaempferol, esculin, robinetin, and okanin were found to be the potential drug candidates to treat male infertility. Molecular dynamics simulation showed tricin as a strong inhibitor of all selected receptor proteins because the ligand–protein complexes remained stabilized during the entire simulation time of 100 ns. Further, an in vivo study was designed to evaluate the effect of tricin in male rats with nicotine-induced infertility. It was explored that a high dose of tricin significantly reduced the levels of alanine transaminase, aspartate transaminase, urea, creatinine, cholesterol, triglyceride, and low-density lipoprotein and raised the level of high-density lipoprotein in intoxicated male rats. A high dose of tricin also increased the reproductive hormones (i.e., testosterone, luteinizing hormone, follicle-stimulating hormone, and prolactin) and reduced the level of DHEA-SO4. The phytochemical (tricin, 10 mg/kg body weight) also showed significant improvement in the histo-architecture after nicotine intoxication in rats. From the current study, it is concluded that the phytochemical tricin could serve as a potential drug candidate to cure male infertility.

## 1. Introduction

Male infertility is a global health issue that affects about 7% of the male population [1]. Various genetic, behavioral, and physical abnormalities are documented as leading causes of male infertility. Furthermore, excessive stress, overdose of drugs, environmental hazards, medical illness, and poor health conditions are also reported in many cases of infertile males [2]. Spermatogenesis and steroidogenesis are the two main processes of testes, through which spermatogonia undergo cell division to transform into mature spermatozoa [3]. The hypothalamic-pituitary-gonadal axis regulates the spermatogenesis induction and persistence of sperm production. The gonadotropin-releasing hormone (GnRH) controls the regulation and release of testosterone, follicle-stimulating hormone (FSH), and luteinizing hormone (LH), which support the spermatogenesis. Any imbalance in GnRH release is directly linked to impaired sperm production [1]. Genetic mutations are heterogenous, which cause chromosomal structural abnormalities in X and Y chromosomes because of deletion of a specific region, copy number variation, the addition of X chromosomes in men, and translocations (Robertsonian or reciprocal), which are the most common events among all the infertile men compared to normal population [4]. The aberrant expression of different proteins governs different physiological dysfunctions in infertile patients [5].

Sex hormone-binding globulin (SHBG) is a multifunctional glycoprotein that is synthesized by the liver, and it binds to steroid hormones and mediates hormonal signaling. The genetic mutations in SHBG genes, more specifically mutated rs6259 and rs727428 loci, are involved in male infertility [6]. SHBG transports and selectively binds to sex hormones to control plasma levels of bioactive sex hormones and to modify their bioavailability. The loss of testosterone bioavailability due to SHBG leads to severe consequences, including male infertility, prostate cancer, and gonadal dysfunction [7].

A disintegrin and metalloproteinase 17 (ADAM17) (also known as TACE) is a membrane-bound protein and is involved in the regulation of shedding of the ectodomain, which inhibits or activates cellular signaling associated with various inflammatory disorders. The expression of ADAM17 is mandatory for the induction of apoptosis in germ cells. Clinically, ADAM17 has been targeted to prevent apoptosis in germ cells with testicular torsion. Testicular torsion is the most common urological illness among young adults, which further prolongs testicular damage in males. Testicular torsion disrupts normal blood flow in testicular tissues, which leads to severe ischemia [8]. Deoxyribonuclease I (DNase I) is a calcium-magnesium-dependent endonuclease associated with DNA fragmentation during apoptosis. Different clinical investigations have reported the involvement of DNase I in induced sperm DNA fragmentation. The DNase I inhibitors have clinically proved their significance in the presentation of sperm fragmentation to treat male infertility [9].

Despite much advancement in the medical field, about 50% of infertility cases are still accountable as idiopathic. Due to a variety of mutations and up- and down-regulation of different proteins in infertility cases, it is still difficult to navigate the exact problem behind every clinical case. The literature provides some of the most reported genes, which, when expressed into proteins, lead to their critical roles in idiopathic infertility in males. Despite the fact that the precise causes are yet not fully understood, genetic, developmental, physical, environmental, and lifestyle variables could all be causal links between infertility and normal health. It has been demonstrated that several genetic variants in infertile males with idiopathic oligoasthenoteratozoospermia are connected to poor spermatogenesis. In the current study, we, therefore, targeted the three most reported proteins because the impaired nature of these proteins plays a crucial role in progressing defective spermatogenesis. Despite the existence of synthetic drugs, we have explored the positive side of natural phytochemicals as natural fighters counter to SHBG, ADAM17, and DNase I as receptor proteins in this study.

## 2. Results

A total of 210 phytochemicals from different plant sources were docked counter to three receptor proteins (i.e., SHBG, ADAM17, and DNase I). The top five ligands against each receptor protein were selected on the basis of ligand–protein interactions through active amino acids of the catalytic pockets and minimum energy structures (Table 1).

### 2.1. Interaction Analysis

**Figure 1:**
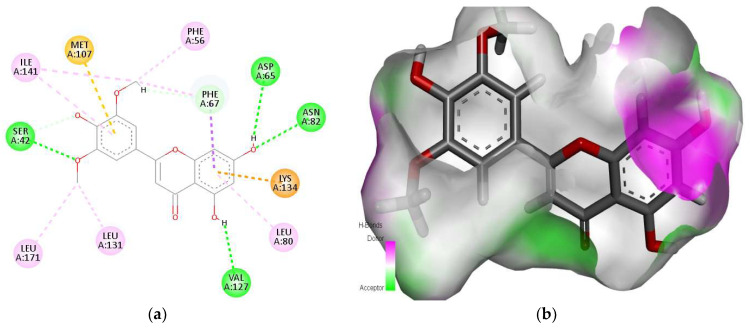
Interaction (**a**) and binding pattern (**b**) of tricin with sex hormone-binding globulin as the receptor. The AutoGrid dimensions between ligand and sex hormone-binding globulin target protein atoms are grid center X: −12.5380, Y: 2.1753, Z: 6.2993 with number of points X: 111, Y: 120, Z: 118, and with spacing (Angstrom): 0.3750.

Against sex hormone-binding globulin (SHBG), the phytochemical tricin with a docking score of −11.09 kcal/mol showed strong interactions with amino acids (i.e., Ser42, Phe56, Asp65, Phe67, Leu80, Asn82, Met107, Val127, Leu131, Lys134, Ile141, and Leu171) of the binding pocket (Figure 1a). The ligand was found to be perfectly fitted into the pocket of the SHBG receptor, shown as a hydrogen bond donor/acceptor surface (Figure 1b). The amino acids Ser42, Asp65, Asn82, and Val127 were involved in conventional H-bonds with tricin. The amino acids Phe56, Leu80, Leu131, Ile141, and Leu171 were found to be responsible for alkyl and Pi–alkyl interactions. A Pi–cation interaction was also found between the ligand and the protein through Lys134. The amino acids Phe67 and Met107 were found to be involved in carbon–hydrogen and Pi–sulfur bonds with the ligand. The ligands myricetin, quercetin, malvidin, and rhamnetin also exhibited strong interaction with the SHBG protein and displayed docking scores of −10.72 kcal/mol, −9.70 kcal/mol, −9.65 kcal/mol, and −8.07 kcal/mol, respectively (Figures S1–S4). The amino acids Lys134 and Arg135 were found to be common residues in these interactions.

**Figure 2:**
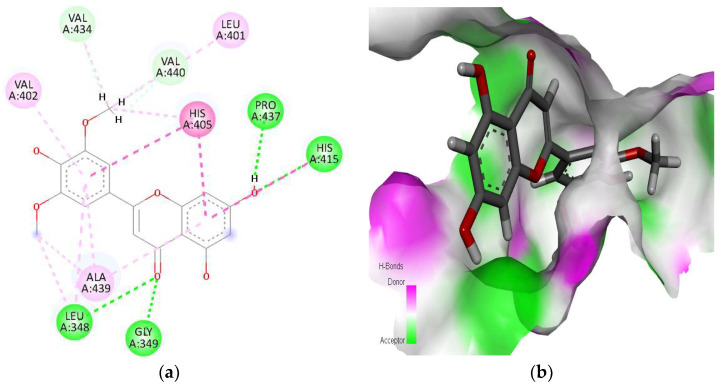
Interaction (**a**) and binding pattern shown as hydrogen bond donor/acceptor surface (**b**) of tricin with disintegrin and metalloproteinase 17 (ADAM17) as the receptor. The AutoGrid dimensions between ligand and disintegrin and metalloproteinase 17 target protein atoms are grid center X: 3.0931, Y: 44.7548, Z: 36.2180 with number of points X: 37, Y: 51, Z: 39, and with spacing (Angstrom): 1.2375.

The third protein, ADAM1 (A disintegrin and metalloproteinase 17), showed interactions with a selected library of ligands. Among the docked compounds, tricin was found to be the best one, with a docking score of −13.15 kcal/mol (Figure 2). Tricin interacted with amino acids Leu348, Gly349, Leu401, Val402, His405, His415, Val434, Pro437, Ala439, and Val440 of the binding pocket of ADAM17. In these interactions, four conventional H-bonds (i.e., Leu348, Gly349, His415, and Pro437), three alkyl and Pi–alkyl bonds (i.e., Leu401, Val402, and Ala439), two carbon–hydrogen bonds (i.e., Val434 and Val440), and one Pi–Pi stacked or Pi–Pi T-shaped (i.e., His405) interactions were found. The phytochemicals callistephin, gallic acid, rosavin, and moronic acid also revealed sufficiently strong interactions with the active site residues of ADAM17 with docking scores of −10.68 kcal/mol, −9.21 kcal/mol, −9.14 kcal/mol, and −9.07 kcal/mol, respectively (Figures S5–S8). The amino acids Ala439 and Val440 were revealed to be common residues among the interactions of the top five phytochemicals with ADAM17.

**Figure 3:**
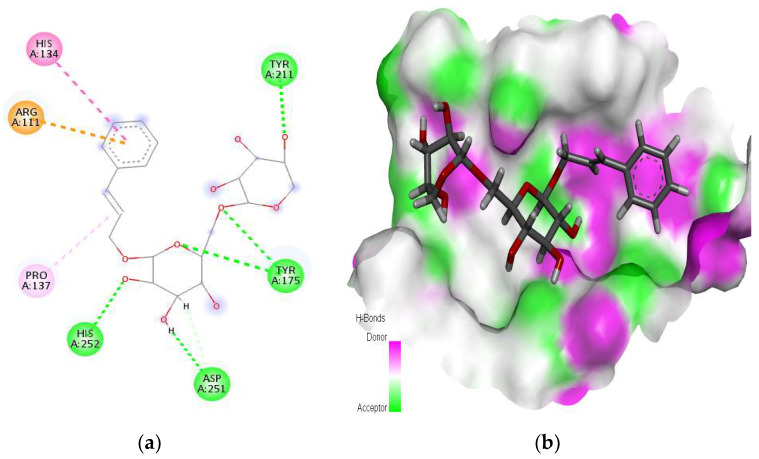
Interaction (**a**) and binding pattern shown as hydrogen bond donor/acceptor surface (**b**) of rosavin with deoxyribonuclease I (DNase I) as the receptor. The AutoGrid dimensions between ligand and deoxyribonuclease I target protein atoms are grid center X: 3.0733, Y: 3.7716, Z: 11.9866 with number of points X: 49, Y: 49, Z: 50, and with spacing (Angstrom): 1.0050.

For the next protein, deoxyribonuclease I (DNase 1), rosavin was revealed as the best candidate to interact with active site residues (i.e., Arg111, His134, Pro137, Tyr175, Tyr211, Asp251, and His252) of the binding pocket of DNase I with docking score of −9.46 kcal/mol (Figure 3). Among these interactions, Tyr175, Tyr211, Asp251, and His252 were involved in conventional H-bonds; His134 underwent a Pi–Pi T-shaped interaction, while Arg111 was found to be involved in a Pi–cation interaction. The amino acid Asp251 was also involved in a carbon H-bond with the ligand. Among the other four phytochemicals, tricin, esculin, okanin, and naringin were found with binding scores of −9.23 kcal/mol, −8.55 kcal/mol, −8.00 kcal/mol, and −7.82 kcal/mol, which also showed significantly strong interactions with residues of the active site of DNase I (Figures S9–S12). The amino acids His134 and Asp251 were found to be common residues among interactions of these ligands with the DNase I protein.

### 2.2. Drug Scanning

SwissADME tool is used to reveal the most suitable drug compounds that follow Lipinski’s rule of five (Ro5), which states that the molecular mass should be <500 Dalton, the hydrogen bond donor should be ≤5, the hydrogen bond acceptor should be ≤10, molar refractive index should be in the range of 40–130, and partition coefficient (LogP) should be ≤5. The purpose of these parameters is to distinguish the drug-like candidates that are suitable to be recommended as leading drug candidates. Among the best-selected phytochemicals against three receptor proteins, myricetin, callistephin, and rosavin violated only one rule. Based on other parameters, the compounds that violate no or one rule can be accepted as drug candidates because of their nontoxic behavior. Moronic acid violated two rules of Ro5, and naringin violated four of Lipinski’s rules, and, therefore, they were excluded from further assessment (Table 2).

For further evaluation, all the best-selected phytochemicals were subjected to ADMET (i.e., absorption, distribution, metabolism, excretion, and toxicity) profiling to confirm the bioavailability and non-toxicity of these drug candidates using ADMETlab 2.0, an online platform. On the basis of best docking scores and ADMET parameters, most of the phytochemicals revealed strong binding interactions with the receptor proteins, good absorption, and no toxicity. The ADMET profiling of the best-selected phytochemicals is given in Table 3.

The ADMET profiling of the best-selected phytochemicals were also performed using another online tool, pkCSM, and the results are given in Table S1. From the results, it was revealed that most of the phytochemicals have high GI absorption and do not cross the blood–brain barrier. They are also not AMES toxic, skin sensitive, and hepatotoxic. According to medical biochemistry of drugs analysis, most of the phytochemicals were accepted according to different rules (i.e., Lipinski rule, Pfizer rule, GSK rule, and Golden triangle) (Table S2). The GSK rule and the Golden Triangle rejected the phytochemical amorin, while other rules accepted it as a drug on the basis of medical biochemistry. Similarly, the GSK rule rejected callistephin, moronic acid, and rosavin phytochemicals. Moronic acid was also rejected by the Pfizer rule and the Golden Triangle. The synthetic accessibility (SA) score estimates how easily drug-like molecules can be synthesized in various areas of the drug discovery process. According to this score, if a compound has an SA score of <6, then the compound is easy to synthesize. This is a development and validation method which is used to characterize the synthetic accessibility of molecules. The score is between 1–10, where 1 means easy to produce and 10 means very difficult to produce [10]. All the phytochemicals showed an SA score < 6, which shows that these compounds are easy to produce (Table S2).

### 2.3. Molecular Dynamics Simulation Study

Based on the results of molecular docking and druggability analyses, the best phytochemicals were further selected for molecular dynamics simulation study. The phytochemicals quercetin, malvidin, rhamnetin, isorhamnetin, gallic acid, kaempferol, esculin, robinetin, and okanin showed strong binding interactions with the receptor proteins and did not violate any rule of Ro5. The phytochemical tricin was found to be involved in sufficiently strong binding interactions with all selected receptor proteins, and, therefore, it was selected for MD simulation studies. The MD simulation was performed to determine the binding properties of these ligands inside the binding pockets of target proteins in the dynamic state. Schrodinger’s Desmond Module was used to perform MD simulation to reveal the stability of the protein–ligand complex.

**Figure 4:**
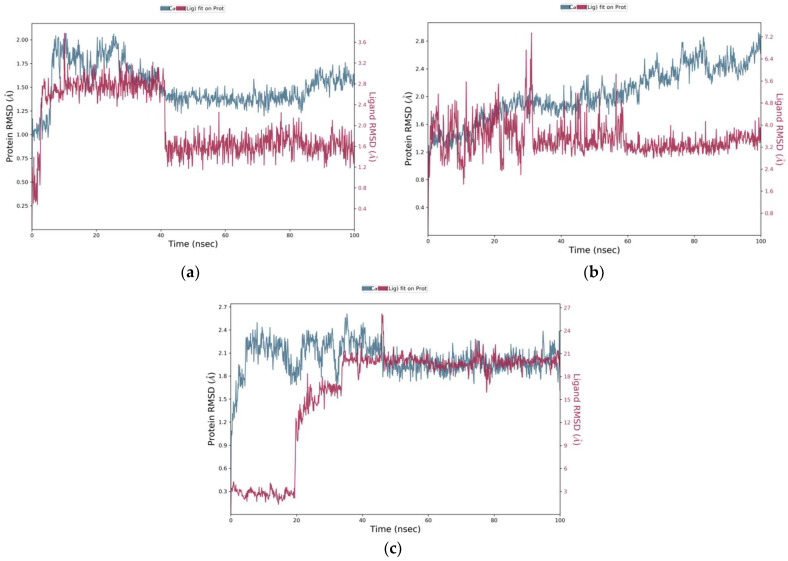
Molecular dynamics simulation showing root mean square deviation trajectories. The MD simulation was run for 100 ns at 1.00314 bar pressure, 310 K temperature, 1000 frames, and 7.4 pH). The MD simulation of the docked complex of (**a**) the tricin–SHBG protein, (**b**) the tricin–ADAM17 protein, and (**c**) the tricin-DNase I protein. The left *Y*-axis (blue) shows the variation of the RMSD of proteins through time. The right *Y*-axis (red) shows the variation of RMSD of tricin phytochemicals through time.

The tricin–SHBG protein complex showed stability via MD simulation. Initially, some minor fluctuations in the protein RMSD were observed, and the value was increased from 1 Å to 2 Å, but the average P-RMSD change was ~1 Å, and the protein was found to be stable throughout the simulation time of 100 ns. The ligand tricin was stabilized after 4 ns and remained firmly attached to the protein SHBG. A mode shift was observed at 40 ns; the ligand again stabilized until the end of the simulation time of 100 ns (Figure 4a). The average change in the ligand RMSD was observed to be ~1.6 Å, which is quite acceptable. For the tricin–ADAM17 protein complex, the mean value of the protein backbone of ADAM17 was found to be 1.2 Å. A change in protein RMSD value up to 3 Å is acceptable. Although some fluctuations were observed in the ligand RMSD at 10 ns, 20 ns, and 28 ns, overall, the RMSD trajectory of tricin showed that the ligand remained stable through a simulation time of 100 ns, and the ligand did not leave its original pocket of ADAM17 protein (Figure 4b). Similarly, in the tricin–DNase I protein complex, the protein RMSD (P-RMSD) provides information about its stability of structural conformation. Changes in P-RMSD from 1.25 to 2.5 Å are acceptable. The ligand showed fluctuations till 32 ns, but it stabilized afterward for the rest of the simulation time and remained firmly bound to the DNase I protein (Figure 4c).

The hydrogen bond interaction stability or the consistency of important amino acids of each receptor protein with the best-selected phytochemical as the ligand throughout molecular dynamics simulation revealed that the ligand complexed with selected respective proteins are stable and could be used as potential candidates in drug designing to cure male infertility (Figures S13–S15). The Root Mean Square Fluctuation (RMSF) analysis of tricin phytochemical complexed with respective proteins exhibited stable peaks throughout the 100 ns MD simulation of important amino acids interacting with the ligand (Figure S16). The findings of these analyses are strong evidence of the stability of best-selected phytochemicals complexed with their respective receptor proteins after molecular docking and MD simulation studies.

**Figure 5:**
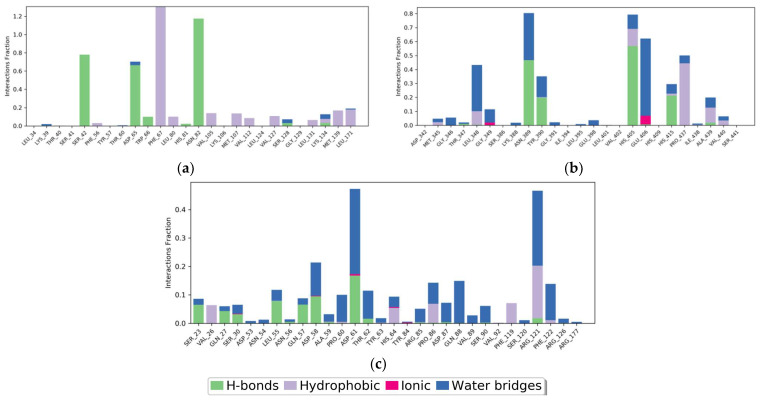
Protein–ligand contact histogram. Protein structures complexed with the best phytochemical; (**a**) SHBG protein complexed with tricin; (**b**) ADAM17 protein complexed with tricin; (**c**) DNase I protein complexed with tricin.

The Protein–Ligand Interaction Fingerprints (PLIF) of the SHBG protein complexed with tricin before and after MD simulation showed that the amino acids Ser42, Asp65, Trp66, and Asn82 are important in stabilizing the complex in drug designing because the residues are involved in making hydrogen bonds with the ligand. The amino acids Phe67, Leu80, Val105, Met107, Val112, Val127, Leu131, Lys134, Met139, and Leu171 were found to be involved in hydrophobic interactions with ligand (Figure 5a). From the PLIF diagram of the ADAM17 protein complexed with tricin, it is revealed that the amino acids Asn389, Tyr390, His405, and His415 (making H-bonds with tricin) are important in stabilizing the complex. The amino acids Leu348, His405, Pro437, and Ala439 are involved in hydrophobic interactions (Figure 5b). In the tricin–DNase I protein complex, the amino acids Ser23, Gln27, Ser30, Leu55, Gln57, Asp58, and Asp61 were found to be important in stabilizing the complex through hydrogen bonding. For hydrophobic interactions, the amino acids Val26, His64, Pro86, Phe119, and Arg121 showed major contributions. In addition, there are a great number of amino acids that are involved in making water bridges with the ligand (Figure 5c).

**Figure 6:**
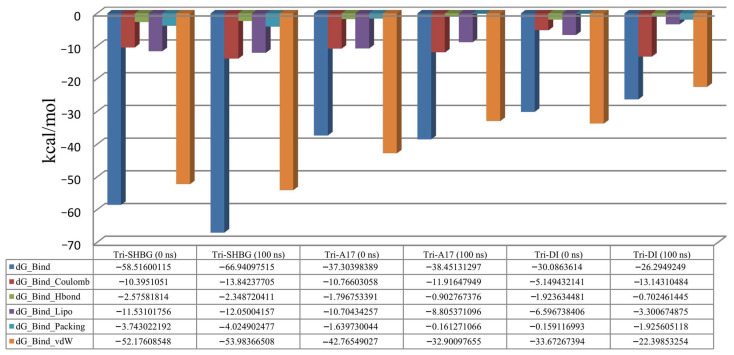
MM-GBSA calculated before and after the simulation. Tri: tricin; A17: ADAM17; DI: DNase I.

For molecular dynamics simulation, the Molecular Mechanics Generalized Born Surface Area (MM-GBSA) was also calculated using the module of Prime MMGBSA v3.000 (Figure 6). Solvation model VSGB and force field OPLS_2005 were employed in this analysis. For the tricin–SHBG protein, the average dG was found to be −63.47 kcal/mol, the dG range was −58.52 to −66.95, and the standard deviation was determined to be 5.98. The average dG for the tricin-ADAM17 protein was found to be −39.57 kcal/mol, with a range of −37.31 to −38.45, and the standard deviation was found to be 4.42. Similarly, the average dG for the tricin–DNase I protein was −24.07 kcal/mol, and the range of −30.09 to −26.29, and the standard deviation was determined to be 5.88. It was revealed that most of the residues of all selected receptor proteins near their binding sites of the ligand contributed significantly to the stabilization of the ligand, which is seeded by lower binding energy values.

### 2.4. In Vivo Evaluation

#### 2.4.1. Liver Function and Lipid Profile

A ‘liver function test’ is a measurement of the liver’s proper functioning and an increase in blood aminotransferases such as ALT and AST, urea, and creatinine activity, indicating dysfunctional liver cells. Elevated ALT and AST values in the blood suggest impaired hepatocyte damage [11]. The results of liver function tests, including ALT and AST, urea, and creatinine concentration, are represented as Mean ± SEM in Table 4 for comparing significant group mean differences of studied animals. According to the data analysis, nicotine intoxication elevated the ALT level considerably (*p* < 0.01) in male rats, i.e., 113.8 ± 5.38A from the range of normal in the control rat group as 46.2 ± 4.11D. At the same time, ALT levels reverted to normal following administration of a high dosage of phytochemical tricin (10 mg/kg b.w.) in groups of male rats at 35 ± 3.27E, respectively. A significant (*p* < 0.01) difference in ALT level was revealed in the plant phytochemical treatment group compared to the control group of female rats. Significantly (*p* < 0.05) elevation in AST, urea, and creatinine levels were reported in intoxicated groups, i.e., 125.8 ± 6.55A, 54 ± 4.90A, and 1.3 ± 0.08A compared to their respective control group rats. At the same time, there was a significant difference in all of the phytochemical-treated groups of rats (male), i.e., 26 ± 3.27E, 17 ± 0.82DE, and 0.8 ± 0.13BC, significant (*p* < 0.01).

Values are mean ± SE (standard error) of the study groups. Different letters (A–F) in superscripts in the same row indicate significant mean differences according to Tukey’s pairwise comparison. The values that do not share a letter (superscript) are significantly different.

In the context of lipid profile, the cholesterol level was also raised in the intoxicated group to 198 ± 9.80A compared to the normal level in the control group (i.e., 152 ± 5.72DE). A high dose of tricin significantly reduced the level of cholesterol by about (138 ± 4.90F) even compared to the positive control group in which the testosterone drug was used. In comparison, the level of TG and LDL were raised to 221 ± 16.33A and 87 ± 2.45A in the nicotine-administrated group, but tricin-treated rats significantly reduced this level to 137 ± 5.32DE and 78 ± 2.53F. HDL cholesterol is also called good cholesterol. Tricin phytochemicals increased the level of HDL, i.e., 42 ± 2.45ABC from 35 ± 2.45F, which was analyzed in the intoxicated group in which nicotine was used for intoxication.

#### 2.4.2. Reproductive Hormones

**Figure 7:**
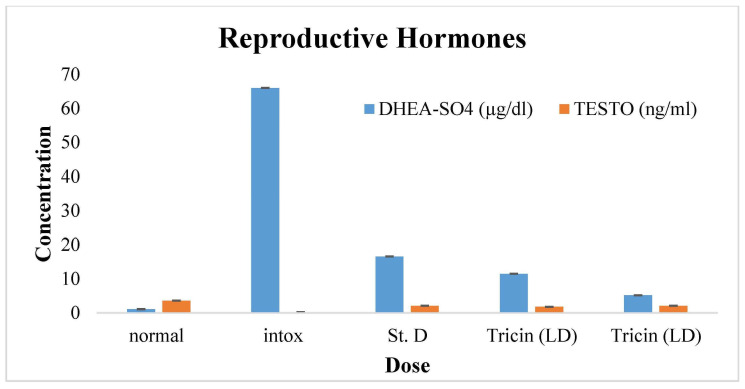
Bar graph depicting the potential of phytochemical (tricin) control treatments on the reproductive hormone response of male rats: Blue bars are indicating DHEA-SO4 test results and orange color bars are indicating testosterone test results. The findings are shown as Means (bars) ± SD (lines). The (*p* < 0.05) is regarded as statistically significant, whereas (*p* < 0.01) is considered very significant.

Testosterone and its potent metabolite dihydrotestosterone (DHT) are the major circulating androgens, mainly in males. The effect of tricin and control treatments on the reproductive hormones of male rats is given in Table 5 and Figure 7. LH is the main stimulant of testosterone from the anterior pituitary, while FSH acts on the testes for spermatogenesis. In the current study, the concentration of testosterone was reduced by about 0.2 ± 0.01D compared to the level that was observed in 3.6 ± 0.02A, but the high dose restored this level from 0.2 ± 0.01D to 2.1 ± 0.01B. Similarly, the levels of LH, FSH, and prolactin were reduced in the negative control group or the intoxicated group (i.e., 0.2 ± 0.01E, 0.3 ± 0.04E, and 0.1 ± 0.01D) compared to male rats of the normal group. The high dose of the tricin phytochemical caused significant increments in these levels of about 0.5 ± 0.02C, 0.53 ± 0.02C, and 0.5 ± 0.02A from intoxicated levels. A high level of DHEA-SO4 decreases the quality and quantity of the sperm. It might also contribute to testicular shrinkage. Likely, nicotine-caused increments in the level of DHEA-SO4 were observed in the intoxicated group as 66.0 ± 3.27A compared to the male rats from the normal group (i.e., 1.1 ± 0.10E). Both low and high doses of tricin restored the level of DHEA-SO4 from 66.0 ± 3.27A to 11.5 ± 0.82C, 5.2 ± 0.0 D and showed a significant difference (*p* < 0.01).

#### 2.4.3. Histo-Architecture of Liver and Testicular Tissue

**Figure 8:**
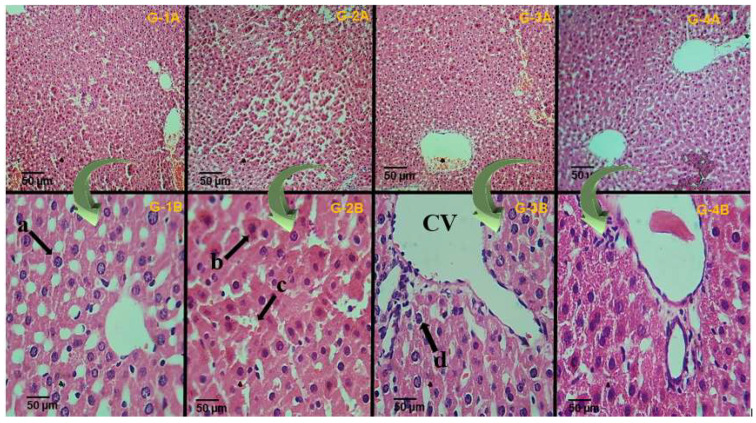
Histological sections obtained from liver tissues of different experimental male rats after staining with an H & E stain (10× and 40×); grouping is the same column-wise. The only difference is in the picture magnification. (**G-1A**,**G-1B**): Normal control group; (**G-2A**,**G-2B**): Intoxicated group; (**G-3A**,**G-3B**): Positive control group; (**G-4A**,**G-4B**): Phytochemical (tricin) treated group. The letters (a–d) indicate different histological features of the liver, such as (a) representing the nucleus, (b) representing the inflammation, (c) representing necrosis, and (d) hepatocytes. (CV) central vein.

The histological examination of liver tissues (seen both in Figure 8 and in Table 6) revealed that the nicotine intoxication of rats significantly damaged the hepatic cells in the hepatic tissues (Figure 8(G-2B)) compared to the normal control group, as a normal hepatic cell is composed of mononuclear mature cells is seen with not any type of necrosis, apoptosis, and inflammation. As edema or liver damage is seen both in the intoxicated or positive control group compared to the plant-treated group in which there is mild inflammation, no edema is seen, and there was no necrosis (Figure 8(G-4B)). The administration of tricin (10 mg/kg body weight (b.w.)) and the positive controls exhibited significant improvement in the histo-architecture after nicotine intoxication (Figure 8(G3B,G4B)).

**Figure 9:**
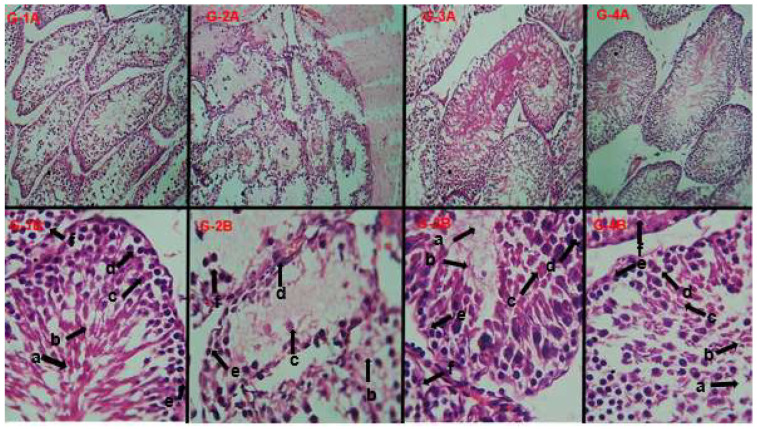
Histological sections taken from testicular tissues of different experimental male rats (10× and 40×); grouping is the same column-wise. The only difference is in the picture magnification. (**G-1A**,**G-1B**): Normal control group; (**G-2A**,**G-2B**): Intoxicated group; (**G-3A**,**G-3B**): Positive control group; (**G-4A**,**G-4B**): Phytochemical (tricin) treated group. The letters (a–f) show different histological features of a testis, including (a) lumen of seminiferous tubule; (b) mature spermatozoa; (c) primary spermatocyte; (d) spermatogonium; (e) seminiferous tubules basement membrane; (f) Leydig cells present between seminiferous tubules spaces.

The histological examination of testicular tissues showed significant damage to the spermatogenic cells by nicotine intoxication. Necrosis was seen in the seminiferous tubules (Figure 9(G-2A,G-2B)) compared to the normal control group (Figure 9(G-2A,G-2B)). Multiple seminiferous tubular cells were found in a normal testis with germinal epithelium of 4 to 6 layers at different spermatogenic stages with mature spermatozoa in the central lumen. Other cells, such as Sertoli and Leydig cells, were also observed in sufficient numbers in the normal testis (Figure 9(G-2A,G-2B)). A noticeable improvement in the histo-architecture was observed in the sections from phytochemical (tricin) administered rats and positive controls after nicotine intoxication (Figure 9(G-3,G-4)). The histological features of testicular cells seen from the testes tissue of male albino rats are given in Table 7.

## 3. Discussion

Infertility is a significant cause of anxiety and depression in society. Different reports have shown the involvement of 20–70% of the male community as part of this leading problem. Despite much advancement in medical science, male infertility is still a leading problem in Asian and European countries due to the involvement of many genetic, physical, and environmental factors. The reduction of oxidative stress in spermatozoa and normal spermatogenesis is key to the end of male infertility [12]. The sperm analysis of infertile men reveals asthenozoospermia due to the down-regulation of many factors or proteins, which are involved in normal signaling pathways. The aberrant expression of metabolic proteins, oxidants, and ROS is mainly found in the seminal plasma of patients with oligoasthenozoospermia [13]. Proteomics and metabolomics are emerging tools that help to overcome the limitations that are not detectable at the laboratory level from the semen analysis. These include different tools and software to identify proteins and peptides in spermatozoa, sperm heads, and testicular tissues to point out the alterations in the level of expression and cellular signaling [14].

In the current study, an extensive literature study was performed to explore the most reported plant-derived phytochemicals, which are in practice against different diseases with potential outcomes. In total, 210 ligands were selected for this research and targeted against sex hormone-binding globulin (SHBG), ADAM17, and DNase I as receptor proteins to manage male infertility due to the involvement of these proteins in male infertility events. Ramgir et al. [12] suggested the use of different medical herbs in suspension form to improve testosterone levels, sperm quality, reduce oxidative stress and enhance the immune system. The administration of decoction of different medicinal plants improves the sperm count, sperm antibody count, and index. A great number of medicinal plants, such as *Coccinia indica*, *Chlorophytum borivilianum*, *Satureja montana* L., *Zingiber officinale* Roscoe, and *Dracaena arborea* have shown significant potential in many clinical investigations to manage the male reproductive damage.

From the current docking study, the phytochemical tricin with a docking score of −11.09 displayed significant binding interactions with Ser42, Phe56, Asp65, Phe67, Leu80, Asn82, Met107, Val127, Leu131, Lys134, Ile141, and Leu171 residues of the active binding pocket of SHBG protein. The other compounds, myricetin, quercetin, malvidin, and rhamnetin, also displayed good binding scores, displaying strong interactions with SHBG protein. The drug assessment of all these predicted candidates fully favored the potential of these phytochemicals as drug candidates. In a study, Esther et al. [15] docked 47 phytocompounds against SHBG. Among all studied phytochemicals, chlorogenic acid with a binding energy of −47.869 kcal/mol and glide docking XP score of −7.255 kcal/mol showed strong interactions with SHBG protein, which showed the potential of natural compounds as drug candidates for the treatment of male infertility. They also performed molecular dynamics simulation for 10 ns to check the stability of chlorogenic acid with the receptor protein and revealed that the ligand was firmly bound with the SHBG target protein for the simulation time.

A disintegrin and metalloproteinase 17 (ADAM17) serves as a metalloprotease that plays a crucial role in a number of cellular signaling pathways. In the current study, we also targeted ADAM 17 active catalytic sites. The phytochemical tricin with a docking score of −13.15 kcal/mol showed strong interactions with Leu348, Gly349, Leu401, Val402, His405, His415, Val434, Pro437, Ala439, and Val440 residues of the binding pocket. The other compounds (i.e., callistephin, gallic acid, rosavin, moronic acid) also interacted with Ala439 and Val440 as common residues of the active site. This suggested the potential of natural bioactive compounds as leading candidates for targeting the ADAM17 protein to cure male infertility. In a study, Mohamed et al. [8] used vitamin D3 to reverse the deteriorating effect of testicular injury by targeting miRNA145 and ADAM17 proteins. The findings of their study supported the ability of vitamin D3 through interactions with the active site of the ADAM17 protein via hydrophobic and hydrophilic interactions with good binding scores. The study suggested the therapeutic use of vitamin D3 to ameliorate testicular damage by targeting the ADAM17 protein.

DNase1, being an endonuclease, regulates the induced apoptotic-based DNA fragmentation. The percentage of damaged DNA is more common in infertile men compared to fertile ones. The DNase1-assisted DNA damage of sperm is the main cause of a defective male reproductive system [16]. In the current study, rosavin with a docking score of −9.46 kcal/mol showed the inhibitory potential to target the active site residues (i.e., Arg111, His134, Pro137, Tyr175, Tyr211, Asp251, and His252) of DNase1 as the target protein. The drug scanning and ADMET profiling displayed the drug-like behavior of this phytochemical as a potent inhibitor of DNase1. Ilić et al. [9] also presented the docking of ascorbic acid as a potent inhibitor of DNase1 for the treatment of male infertility. Ascorbic acid formed H-interactions with Asp168 and Asn170 of the binding pocket. The results indicated the inhibitory potential of ascorbic acid to target the induced apoptosis of sperm DNA. In another study [17], a computational approach was used to compare the effectiveness of reported bioactive chemicals in plant hemp towards three proteins associated with male fertility (i.e., 24-dehydrocholesterol reductase (DHCR24), cation channel sperm associated 1 (CatSper 1), and testis expressed protein 11 (TEX 11)) with clomiphene, a particular estrogen-receptor stipulated for the medical management of infertility. A large number of compounds showed commendable binding affinity, according to the analyses of protein modeling, docking simulations, ADME toxicity, induced-fit docking simulation, as well as linking free energy over ligand–protein complexes. These findings corroborated our findings that plants contain phytochemicals that inhibit proteins involved in testicular damage and male infertility.

The drug scanning and ADMET profiling of all the best-selected compounds displayed the maximum potential of these compounds as drug candidates. Almost all selected top ligands followed all five of Lipinski’s rules (Ro5), except naringin, which violated four rules of the Ro5, and moronic acid, which violated two rules of the Ro5. The ADMET scanning further proved the potential of these compounds because they were found to be nontoxic and possessed good bioavailability. The molecular docking, druggability analyses, and molecular dynamics simulation studies revealed tricin, quercetin, malvidin, rhamnetin, isorhamnetin, gallic acid, kaempferol, esculin, robinetin, and okanin phytochemicals as potential drug candidates for the management of male infertility. These phytochemicals also followed all druggability parameters for being good or potential drug candidates. These ligands displayed no Ames toxicity and carcinogenicity. On the basis of the SA score, Lipinski’s rule, Pfizer rule, GSK rule, and the Golden Triangle, the medical biochemistry profiling of the best-selected phytochemicals (i.e., tricin, malvidin, quercetin, gallic acid, and esculin) also confirmed that these could be used as drug candidates in drug discovery processes against male infertility.

In order to investigate the therapeutic effects of phytochemical tricin on the reproductive hormones employing male albino rats as experimental animals, the present investigation was carried out. Increased serum liver enzyme concentrations in blood flow can be caused by the degradation of toxic phytoconstituents found in plants and byproducts, which are expelled by the liver and may alter the normal functioning of the liver [18]. A liver function test measures how well the liver is working, and an increase in the level of activity of blood aminotransferases, such as ALT and AST, suggests that the liver cells are not operating properly. Increased ALT and AST levels in the blood are indicative of damaged hepatocytes [11]. The relative hepatoprotective efficacy of Sharbat-e-Deenar (SD) and Majoon-e-Dabeed-ul-ward (MD) toward CCl_4_-induced liver toxicity was examined by Shakya et al. [19]. The excretion of hepatic enzymes, such as AST, ALT, LDH, and SALP in blood, as well as the peroxidation of lipids in the tissue of the liver, were shown to be considerably increased (*p* < 0.05) by CCl_4_ exposure; however, these variables were all reduced by the application of MD and SD. According to Uemura et al. [20], dyslipidemia is characterized by an upsurge in the plasma levels of cholesterol or triglycerides (TGs) or both, as well as a reduction in high-density lipoprotein (HDL) levels.

The main hormones impacting erectile functions in men and regulating libido in women include gonadotrophins (FSH and LH), testosterone, and prolactin [21]. By screening powerful phytochemicals, Biswas et al. [22] studied integrated computational techniques for suppressing sex hormone-binding globulin in infertility among men. With binding affinity values of −9.2, −9.0, and −8.8 kcal/mol, correspondingly, dorsilurin E, cryptomisrine, and isoiguesterin have been shown to be promising SHBG inhibitors by computational screening and molecular docking investigations. The research also showed greater binding affinities compared to the control medication, anastrozole (−7.0 kcal/mol), which is consistent with the results of the research. They also considerably (*p* < 0.01) increased the level of male reproductive hormones. The phytochemical composition, antioxidant activity, and androgenic effects associated with four South African herbal products were studied by Masuku et al. [23]. When contrasted with the control, it was determined that *T. sambesiaca* methanol extract possessed the greatest amount of testosterone production capacity, while *P. africanum* and *T. sambesiaca* acetone extracts had significantly higher testosterone production at different doses, respectively. In individuals with metabolic syndrome, Usharani et al. [24] assessed the impact of a standardized aqueous extract of *Phyllanthus emblica* fruit on lipid profile and oxidative stress. It was concluded that after 12 weeks, there was a significant (*p* < 0.001) mean percentage change for HDL-C (+7.33%, +22.16%, *p* < 0.05), TC (−7.71%, −11.11%), TG (−9.81%, −19.22%), and LDL-C (−11.39%, −21.8%), respectively, which are in accordance with our results of this study.

Male erections are influenced by reproductive hormones, such as testosterone, gonadotrophins, and prolactin [25]. The hypothalamo–pituitary gonadal pathway closely controls reproduction, and gonadotrophin-releasing hormone (GnRH), through the hypothalamus, influences gonadotrophin secretion, LH, and FSH via the anterior pituitary. While LH plays an important part in the generation and discharge of testosterone from Leydig cells, which is crucial for spermatogenesis and the growth and development of the reproductive system, including the testis and prostate, FSH stimulates spermatogenesis. Additionally, it controls the manifestation of secondary sexual traits in men, such as enhanced bone density, muscular mass, endocrine function, and body hair development [26]. The purpose of FSH is to promote spermatogenesis, whereas LH is responsible for promoting testosterone production and release. Testosterone influences a greater flow of blood, which in turn encourages the development of the target tissue. The results of the current study are in agreement with those of Munir et al. [27], who also noted that CCl_4_ administration caused testicular toxicity in rats and described how it led to germinal layer degeneration, decreased serum levels of LH and FSH and testosterone in male rats. The reduction in testosterone secretion may be caused by nicotine’s direct effect on Leydig cells, which renders them insensitive to LH and FSH and impairs the production of male reproductive hormones. In a different study, Kubba et al. [28] explored the possible benefits of *Borago officinalis* methanolic extract on albino male mice testes and spleen either separately or following the combination of both extracts of plants with CCL_4_. The findings showed that ameliorative consequences were achieved by increasing luminal and interstitial space, recovering the structure of seminiferous tubules and spermatozoa, and increasing the total number of spermatocytes and spermatids in comparison with the administered groups.

This current study demonstrated the beneficial effects of tricin phytochemicals on the structural makeup as well as endocrine functions of the male reproductive system. The specialized phytochemical tricin (3′,5′-dimethoxyflavone) not only provides stress tolerance in plants but is also involved in defense responses. It has a wide range of pharmaceutical applications, such as anti-inflammatory, anticancer, antioxidant, antiviral, and antihistaminic properties, and, therefore, is a promising nutraceutical [29,30].

## 4. Materials and Methods

### 4.1. Retrieval and Optimization of Phytochemicals

An extensive literature survey was performed to collect phytochemicals from different medicinal plants. In total, 210 phytochemicals were taken as ligands, and their chemical structures were downloaded from the PubChem database in SDF format [31]. Using the Open Babel tool of PyRx software (v0.8) [32], the ligand molecules were prepared after the minimization of their energies following their conversion into a pdbqt format for use in the molecular docking study.

### 4.2. Retrieval and Preparation of Receptor Proteins

A total of three proteins were used in this study as receptor or target proteins. The three-dimensional (3D) structures of sex hormone-binding globulin (PDB ID: 6PYF), A disintegrin and metalloproteinase 17 (PDB ID: 2I47), and deoxyribonuclease I (PDB ID: 4AWN) were retrieved from the Protein Data Bank [33]. The receptor proteins were first prepared for molecular docking study by removing solvent and already bound ligand molecules, adding hydrogen atoms, adjusting partial charges, 3D protonation, and minimizing energy using Molecular Operating Environment (MOE) software (v2014.0901) [34].

### 4.3. Molecular Docking Study

The molecular docking analysis was conducted between plant-derived phytochemicals and three different receptor proteins. The PyRx software was employed to conduct a molecular docking study. The prepared structures of receptor proteins were imported into PyRx and converted into a pdbqt format. A default exhaustiveness value of 8 was used to maximize the ligand–protein binding conformational investigations. The docked conformations with the lowest docking score and root mean square deviation (RMSD) were selected as the best ones [35]. Finally, to visualize the interactions between ligand and receptor proteins, the Discovery Studio software (v21.1.0.20298) was used [36].

### 4.4. Druggability Analyses

The online tool SwissADME [37] was used to evaluate the drug-like behavior of the selected ligands to check if they follow Lipinski’s rule of five (Ro5). The candidates that followed Lipinski’s drug parameters were selected for further drug assessment. The ADMET properties (i.e., absorption, distribution, metabolism, excretion, and toxicity) of the selected drug-like compounds were analyzed using the ADMETlab 2.0 [38], pkCSM [39], and SwissADME [37] online platforms.

### 4.5. Molecular Dynamics Simulation Study

The molecular dynamics (MD) simulation study was employed for the verification of the stability of docked complexes (i.e., ligand–protein complexes). In this study, complexes were selected for MD simulation on the basis of their minimum docking score, lowest RMSD, minimum violations of Lipinski’s Ro5 (i.e., zero or one), and druggability analysis. To perform MD simulations, the Schrodinger’s Desmond Module was used [40]. For making predictions, a water-soaked solvent solution was employed. To resolve problems related to MD simulations, the TIP3P water model was employed. For creating an orthorhombic simulation, a box was used with periodic boundary conditions along with a buffer distance from the outer surface of the protein of at least 10 Å. A solution of NaCl (0.15 M) was added to maintain the isosmotic state of the simulation box. A suitable number of counter-ions were also used to neutralize the system. Before the production run of the MD simulation, a predetermined equilibration process was followed. The MD simulation was run at 1.013 bar pressure and 310 K temperature. Each simulation was run for 100 ns and recorded 1000 frames in the trajectory. To analyze the MD simulation trajectory, the simulation interactions diagram was used [41].

### 4.6. In Vivo Evaluation

#### 4.6.1. Grouping of Animals and Doses Plan

The animal experiment was reviewed and approved by the Ethics Committee of the Government College University, Faisalabad (approval number: GCUF/ERC/2237). No human participants and/or tissues were involved in this study. The in vivo study was comprised of male albino rats (i.e., 90 to 99 g). A normal husbandry environment was provided to the study animals. The animals were given a normal diet and a 12 h light and dark cycle. The animals used in the study were brought from the Department of Physiology, Government College University Faisalabad, and placed in an ordinary husbandry setting. Five groups of animals were set, with four animals in each group. Group 1 was the healthy control (not any drug administered); Group 2 was the intoxicated control (administered with nicotine 2 mg/kg body weight (b.w.) orally for 15 days before the hormone levels were analyzed—when the level of reproductive hormone was decreased, one group was administered with testosterone as a positive control by injection 10 mg/kg body weight (b.w.) intramuscular (i.m.) (positive control group and Group 3)), and the remaining two groups (namely Group 4 and Group 5) were administered with an aqueous solution of tricin phytochemicals (1% stock solution) and administered in two different doses (i.e., 05 mg/kg body weight (b.w.) and 10 mg/kg (b.w.) to intoxicated male groups for 42 days (7 weeks). Tricin in a pure powdered form was purchased from Sigma Aldrich (CAS No. 22697-65-0, MDL No. MFCD17166988). A normal husbandry environment was provided to all the study animals. For six weeks, the animals were provided a normal diet as well as 12 h of light and dark cycle in the animal house.

#### 4.6.2. Biochemical Determination

The following parameters were determined in blood samples collected from study subjects. Liver enzymes, such as transaminases (i.e., alanine transaminase (ALT) and aspartate transaminase (AST)) [42], creatinine and urea [43], and lipid profiles, including cholesterol, triglyceride (TG), high-density lipoprotein (HDL), and low-density lipoprotein (LDL), were determined through the automated photometric method [44].

#### 4.6.3. Determination of Hormones

Follicle-stimulating hormone (FSH) (to check for menopause), testosterone (to check the deficiency in men), luteinizing hormone (LH), progesterone, prolactin, and dehydroepiandrosterone (DHEA)-SO4 were determined using ELISA kit [45].

#### 4.6.4. Histological Examination of Tissues

The animals were dissected for histological examination, and the tissue samples were collected in a buffered formalin (neutral) container. Sections were stored in labeled tissue cassettes. The method described by Bancroft and Gamble [46] was adopted to process the embedding in paraffin wax, followed by microtomy, which was performed for taking thin sections. After deparaffinization, a Hematoxylin and Eosin (H & E) stain was used to stain the sections (following the standard method described by Bancroft and Gamble), which were then observed under a microscope.

#### 4.6.5. Statistical Analysis

The acquired data were reported as Mean ± SD and analyzed statistically using a one-way ANOVA test [47], and the difference among groups pairwise research was assessed by Tukey’s test and Fisher’s test using statistical software Minitab 17 (trial version).

## 5. Conclusions

Male infertility is a global problem that is related to multiple complications of the male reproductive system, including genetic and physical complications along with environmental factors. Despite much improvement in the medical field, there is still a concept of idiopathic infertility. There is a lack of clinical knowledge of infertility cases, which is the main reason for the low amount of scientific work in this domain. In the current study, three different receptor proteins were targeted, which are directly linked to male infertility events. Out of 210 docked phytochemicals, 15 were selected as top-hit compounds on the basis of their good docking scores and binding interactions with the active site amino acids of three selected receptor proteins (i.e., five top ligands against each receptor). Molecular dynamics simulation revealed the stability of tricin as a ligand in complexes with selected receptor proteins. Further, the drug scanning and ADMET profiling showed the potential of top-selected phytochemicals as leading drug candidates with good efficacy, no carcinogenicity, and cardiotoxicity. Therefore, in the future, these compounds could be used as potential drug candidates against selected receptor proteins involved in male infertility. The in vivo study revealed that herbal plants are comprised of a variety of phytochemicals, and these constituents had significant antioxidant properties both in vitro and in vivo, as well as the ability to significantly restore male reproductive hormones in albino rats as a result of the treatment corresponding to amounts of tricin administered.

## Figures and Tables

**Table 1:** Energy profiling of top phytochemicals as ligands docked to three selected receptor proteins.

Sr. No.	PubChem ID	Phytochemical	Receptor	Docking Score (kcal/mol)	Interacting Residues
1	5281702	Tricin	SHBG (6PYF)	−11.09	Ser42, Phe56, Asp65, Phe67, Leu80, Asn82, Met107, Val127, Leu131, Lys134, Ile141, Leu171
2	5281672	Myricetin		−10.72	Asn61, Asp64, Asp65, Gly129, Lys134, Arg135
3	5280343	Quercetin		−9.70	Asp64, Asp65, His83, Trp84, Ser133, Lys134, Arg135
4	159287	Malvidin		−9.65	Thr60, Asp64, Asn82, His83, Trp84, Ser133, Lys134, Arg135
5	5281691	Rhamnetin		−8.07	Asn61, Asp64, His83, Gly129, Ser133, Lys134, Arg135
6	5281702	Tricin	ADAM17 (2I47)	−13.15	Leu348, Gly349, Leu401, Val402, His405, His415, Val434, Pro437, Ala439, Val440
7	44256621	Callistephin		−10.68	Leu348, Leu401, Val402, His405, Glu406, His409, Pro437, Ala439, Val440
8	370	Gallic acid		−9.21	Glu398, Val402, His405, Val434, Ala439, Val440
9	9823887	Rosavin		−9.14	Gly349, Met345, Leu348, Tyr390, Leu401, Val402, His405, Glu406, Ala439, Val440
10	489941	Moronic acid		−9.07	Met345, Leu350, His405, His409, His415, Ile438, Ala439
11	9823887	Rosavin	DNase 1	−9.46	Arg111, His134, Pro137, Tyr175, Tyr211, Asp251, His252
12	5281702	Tricin		−9.23	Glu39, Tyr6, Arg111, His252
13	5281417	Esculin		−8.55	His134, Asn170, Tyr175, Tyr211, Asp251
14	5281294	Okanin		−8.00	Glu39, Arg41, His134, Asn170, Asp251, His252
15	442428	Naringin		−7.82	Arg111, His134, Pro137, Asn170, Ser174, Tyr175, Asp251

**Table 2:** Drug-like analysis of hit compounds.

Sr.	Ligands	Target	Molecular Weight (<500 Dalton)	HBD (≤5)	HBA (≤10)	LogP (≤5)	Molar Refractivity (40–130)	Violations
1	Tricin	SHBG ADAM17 DNaseI	330.29	3	7	2.15	86.97	0
2	Myricetin	SHBG	318.24	6	8	0.79	80.06	1
3	Quercetin		302.24	5	7	1.23	78.03	0
4	Malvidin		331.30	4	7	0.92	87.13	0
5	Rhamnetin		316.26	4	7	1.63	82.50	0
6	Isorhamnetin		316.26	4	7	1.65	82.50	0
7	Callistephin	ADAM17	468.84	7	10	−1.71	112.12	1
8	Gallic acid		170.12	4	5	0.21	40.47	0
9	Kaempferol		286.24	4	6	1.58	76.01	0
10	Moronic acid		454.7	1	3	6.03	135.69	2
11	Rosavin	ADAM17 DNaseI	428.4	6	10	−1.07	101.30	1
12	Esculin	DNaseI	340.28	5	9	−0.62	78.65	0
13	Robinetin		302.24	5	7	1.12	78.03	0
14	Okanin		288.25	5	6	1.69	76.36	0
15	Naringin		580.53	8	14	−0.87	134.91	4

HBD: Number of hydrogen bond donors, HBA: Number of hydrogen bond acceptors, logP: The logarithm of octanol/water partition coefficient.

**Table 3:** ADMET profiling of the best-selected phytochemicals.

Category	Property	Myricetin	Malvidin	Rhamnetin	Quercetin	Isorhamnetin	Okanin	Callistephin
Absorption	Caco-2 > −5.15	−5.653	−5.159	−5.109	−5.204	−5.056	−5.375	−6.196
	Pgp-Substrate	−−−	++	−−−	−−−	−−−	−−−	+
	Pgp-Inhibitor	−−−	−−−	−−−	−−−	−−−	−−−	−−−
	HIA	−−−	−−−	−−−	−−−	−−−	−−−	++
Distribution	BBB	−−−	−−−	−−−	−−−	−−−	−−−	−−
	PPB	92.77%	93.13%	96.30%	95.45%	96.24%	99.08%	89.62%
Metabolism	CYP1A2-Inhibitor	++	++	+++	+++	+++	+	−−−
	CYP1A2 substrate	−−	++	+	−−	++	−−−	−−−
	CYP2C19 inhibitor	−−−	−−−	−−−	−−−	−−	−−−	−−−
	CYP2C19 substrate	−−−	−−−	−−−	−−−	−−−	−−−	−−−
	CYP2C9 inhibitor	+	−−−	+	+	+	+	−−−
	CYP2C9 substrate	−	++	++	+	++	−	−
	CYP2D6 inhibitor	−−−	−−−	−	−	+	−−−	−−−
	CYP2D6 substrate	−−	+	−	−−	−	−−	−−
	CYP3A4 inhibitor	−−	−−	−−	−	+	−−	−−−
	CYP3A4 substrate	−−−	−−	−−−	−−	−−−	−−	−−−
Excretion	Clearance	7.716	11.093		8.284	6.991	16.361	7.971
Toxicity	AMES	−	−−	+	+	+	++	++
	hERG	−−	−−	−−−	−−−	−−−	−−−	−−−
	FDAMDD	+	+++	−	−−−	−−−	−	−−−
	H-HT	−−−	−−−	−−−	−−−	−−−	−−	−−
Category	Property	Kaempferol	Gallic Acid		Rosavin	Esculin	Robinetin	Tricin
Absorption	Caco-2 > −5.15	−4.974	−5.728		−5.406	−5.950	−5.325	−4.970
	Pgp-Substrate	−−−	−−−		−	−	−−−	+++
	Pgp-Inhibitor	−−−	−−−		−−−	−−−	−−−	−−−
	HIA	−−−	−−−		+++	++	−−−	−−
Distribution	BBB	−−−	−−−		−	++	−−−	−−−
	PPB	97.86%	53.49%		43.91%	56.10%	94.64%	91.48%
Metabolism	CYP1A2-Inhibitor	+++	−−−		−−−	−−−	++	+++
	CYP1A2 substrate	−−	−−−		−−−	−−−	−−	+++
	CYP2C19 inhibitor	−−	−−−		−−−	−−−	−−−	−−
	CYP2C19 substrate	−−−	−−−		−−−	−−−	−−−	−−−
	CYP2C9 inhibitor	+	−−		−−−	−−−	+	+
	CYP2C9 substrate	++	−−−		−−	−	−	++
	CYP2D6 inhibitor	++	−−−		−−−	−−−	−−	+
	CYP2D6 substrate	−−	−−		−−	−−	−−	++
	CYP3A4 inhibitor	+	−−−		−−−	−−−	−	+
	CYP3A4 substrate	−−−	−−−		−−−	−−−	−−−	−−
Excretion	Clearance	6.868	10.108		1.218	4.015	7.773	6.626
Toxicity	AMES	+	−−−		−−	−−	+	−
	hERG	−−−	−−−		−−−	−−−	−−−	−−
	FDAMDD	−−	−−−		−−−	−−−	−	++
	H-HT	−−−	−−		−−	−−	−−	−−−

Pgp: P-glycoprotein; HIA: Human Intestinal Absorption; BBB: Blood–Brain Barrier; PPB: Plasma protein binding. The values are represented with different symbols for prediction probability: 0–0.1 (−−−), 0.1–0.3 (−−), 0.3–0.5 (−), 0.5–0.7 (+), 0.7–0.9 (++), and 0.9–1.0 (+++). The sign ‘+++’ or ‘++’ represents the like hood of toxic or defective, while ‘−−−’ or ‘−’ represents nontoxic or appropriate. Tip: For the classification endpoints, the prediction probability values are transformed into six symbols: 0–0.1 (−−−), 0.1–0.3 (−−), 0.3–0.5 (−), 0.5–0.7 (+), 0.7–0.9 (++), and 0.9–1.0 (+++).

**Table 4:** Potential of tricin phytochemical on liver enzymes of control and experimental groups of male rats.

	Control	Intoxicated	Positive Control	Tricin (LD)	Tricin (HD)
ALT (IU/L)	46.2 ± 4.11 ^D^	113.8 ± 5.38 ^A^	68 ± 4.90 ^C^	89.5 ± 5.32 ^B^	35 ± 3.27 ^E^
AST (IU/L)	51.8 ± 4.11 ^CD^	125.8 ± 6.55 ^A^	55.8 ± 3.30 ^C^	72 ± 5.72 ^B^	26 ± 3.27 ^E^
Urea (mg/dL)	22 ± 1.63 ^C^	54 ± 4.90 ^A^	24 ± 2.45 ^B^	19 ± 0.82 ^D^	17 ± 0.82 ^DE^
Creatinine (mg/dL)	0.8 ± 0.13 ^BC^	1.3 ± 0.08 ^A^	0.7 ± 0.16 ^BC^	0.7 ± 0.16 ^BC^	0.8 ± 0.13 ^BC^
Cholesterol (mg/dL)	152 ± 5.72 ^DE^	198 ± 9.80 ^A^	160.8 ± 7.37 ^CD^	178 ± 8.16 ^B^	138 ± 4.90 ^F^
TG (mg/dL)	173 ± 9.80 ^B^	221 ± 16.33 ^A^	122 ± 8.16 ^EF^	148 ± 7.35 ^CD^	137 ± 5.32 ^DE^
HDL (mg/dL)	38 ± 1.63 ^CDE^	35 ± 2.45 ^F^	45 ± 2.45 ^A^	41 ± 2.45 ^ABCD^	42 ± 2.45 ^ABC^
LDL (mg/dL)	80 ± 2.45 ^E^	87 ± 2.45 ^A^	85 ± 4.08 ^B^	84 ± 3.27 ^CD^	78 ± 2.53 ^F^

**Table 5:** Effect of selected plant phytochemical and control treatments on reproductive hormones of male rats.

	Control	Intoxicated	Positive Control	Tricin (LD)	Tricin (HD)
TESTO (ng/mL)	3.6 ± 0.02 ^A^	0.2 ± 0.01^D^	2.1 ± 0.08 ^B^	1.8 ± 0.01 ^C^	2.1 ± 0.01 ^B^
LH (IU/L)	0.8 ± 0.02 ^A^	0.2 ± 0.01 ^E^	0.6 ± 0.04 ^B^	0.4 ± 0.01 ^CD^	0.5 ± 0.02 ^C^
FSH (IU/L)	0.8 ± 0.04 ^A^	0.3 ± 0.04 ^E^	0.7 ± 0.01 ^B^	0.3 ± 0.01 ^DE^	0.53 ± 0.02 ^C^
PROL (ng/mL)	0.2 ± 0.04 ^C^	0.1 ± 0.01 ^D^	0.3 ± 0.02 ^B^	0.3 ± 0.02 ^B^	0.5 ± 0.02 ^A^
DHEA-SO_4_ (µg/dL)	1.1 ± 0.10 ^E^	66.0 ± 3.27 ^A^	16.6 ± 0.82 ^B^	11.5 ± 0.82 ^C^	5.2 ± 0.01 ^D^

Different letters (A–E) in superscripts in the same row indicate significant mean differences. This is Tukey’s pairwise comparison and showing that values that do not share a letter (superscript) are significantly different.

**Table 6:** Histological features of hepatic cells seen from hepatic tissue of male albino rats.

Group Number	Architecture Intact/Distorted	Necrosis	Periportal Inflammation Acute/Chronic	Congestion	Edema	Apoptosis	Hemorrhage
Control (G-1)	Intact	Not Seen	Not Seen	Not Seen	Not Seen	Not Seen	Not Seen
Intoxicated (G-2)	Intact	Seen	Moderate, chronic++	Not seen	Seen	Seen	Not seen
Standard drug (G-3)	Intact	Not seen	Mild, chronic+	Not seen	Seen	Seen	Not seen
Test group (G-4)	Intact	Not seen	Mild, chronic+	Not seen	Not seen	Seen	Not seen

**Table 7:** Histological features of testicular cells seen from testes tissue of male albino rats.

Group Name	Capsule	Seminiferous Tubules	Sertoli Cells	Germinal Cell Layer	Spermatogenesis	Tubular Necrosis	Edema	Inflammation
Control (G-1)	Intact	Intact	Seen	Seen	Seen	Not seen	Not seen	Not seen
Intoxicated (G-2)	Intact	Altered	Seen	Cellular depletion	Not seen	Seen	Seen	Chronic++
Standard drug (G-3)	Intact	Intact	Seen	Cellular depletion	Diminished	Not seen	Not seen	Mild+
Test group (G-4)	Intact	Intact	Seen	Seen	Seen	Not seen	Not seen	Not seen

## Data Availability

Data are contained within the article and Supplementary Materials.

## Supplementary Materials

The following supporting information can be downloaded at https://www.mdpi.com/article/10.3390/molecules28237693/s1, Figure S1. Interaction (a) and binding pattern (b) of myricetin with sex hormone-binding globulin as the receptor. Figure S2. Interactions (a) and binding patterns (b) of quercetin with SHBG as a receptor protein. Figure S3. Interactions (a) and binding patterns (b) of malvidin with SHBG as a receptor protein. Figure S4. Interactions (a) and binding patterns (b) of rhamnetin with SHBG as a receptor protein. Figure S5. Interaction (a) and binding pattern (b) of callistephin with disintegrin and metalloproteinase 17 (ADAM17) as the receptor. Figure S6. Interactions (a) and binding patterns (b) of gallic acid with ADAM17 as a receptor protein. Figure S7. Interactions (a) and binding patterns (b) of rosavin with ADAM17 as a receptor protein. Figure S8. Interactions (a) and binding patterns (b) of moronic acid with ADAM17 as a receptor protein. Figure S9. Interactions (a) and binding patterns (b) of tricin with DNaseI as a receptor protein. Figure S10. Interactions (a) and binding patterns (b) of esculin with DNaseI as a receptor protein. Figure S11. Interactions (a) and binding patterns (b) of okanin with DNaseI as a receptor protein. Figure S12. Interactions (a) and binding patterns (b) of naringin with DNaseI as a receptor protein. Figure S13. Hydrogen bond interaction stability (consistency) of key residues of SHBG protein with tricin. Figure S14. Hydrogen bond interaction stability (consistency) of key residues of the ADAM17 protein with tricin. Figure S15. Hydrogen bond interaction stability (consistency) of key residues of the DNaseI protein with tricin. Figure S16. Residue-wise Root Mean Square Fluctuation (RMSF) of protein–ligand complexes during 100 ns. (a) Tricin-SHBG protein; (b) tricin-ADAM17 protein; (c) tricin-DNase I protein. The RMSF of docked protein showed no fluctuation of interacting residues, where green vertical lines represent interacting residues. Table S1. ADMET screening through pkCSM. Table S2. Medical biochemistry of drugs.
