# Supplementary material for: Exploring the Potential of Plant Bioactive Compounds against Male Infertility: An In Silico and In Vivo Study
Source: Molecules. 2023 Nov 21;28(23):7693. doi: 10.3390/molecules28237693 (PMC10707554; doi:10.3390/molecules28237693)
Supplement: Supplementary file 1 [file molecules-28-07693-s001.zip › molecules-2659949-supplementary.pdf]

Article

Exploring the Potential of Plant Bioactive Compounds Against Male Infertility: An In Silico and In Vivo Study

Muhammad Jahangeer [1], Ghulam Mustafa [1,*], Naveed Munir [2], Sibtain Ahmed [3,4] and Khalid Mashai Al-Anazi [5]

1 Department of Biochemistry, Government College University Faisalabad, Faisalabad 38000, Pakistan; rajahangeer@gcuf.edu.pk
2 School of Health Sciences, Department of Biomedical Laboratory Sciences, University of Management and Technology, Lahore 54782, Pakistan; naveed.munir@umt.edu.pk
3 Scripps Institution of Oceanography, University of California San Diego, 9500 Gilman Drive, La Jolla, CA 92093, USA; sibtain@ucsd.edu
4 Department of Biochemistry, Bahauddin Zakariya University, Multan 60800, Pakistan
5 Department of Zoology, College of Science, King Saud University, Riyadh 11451, Saudi Arabia; kalanzi@ksu.edu.sa
* Correspondence: drghulammustafa@gcuf.edu.pk

Supporting information

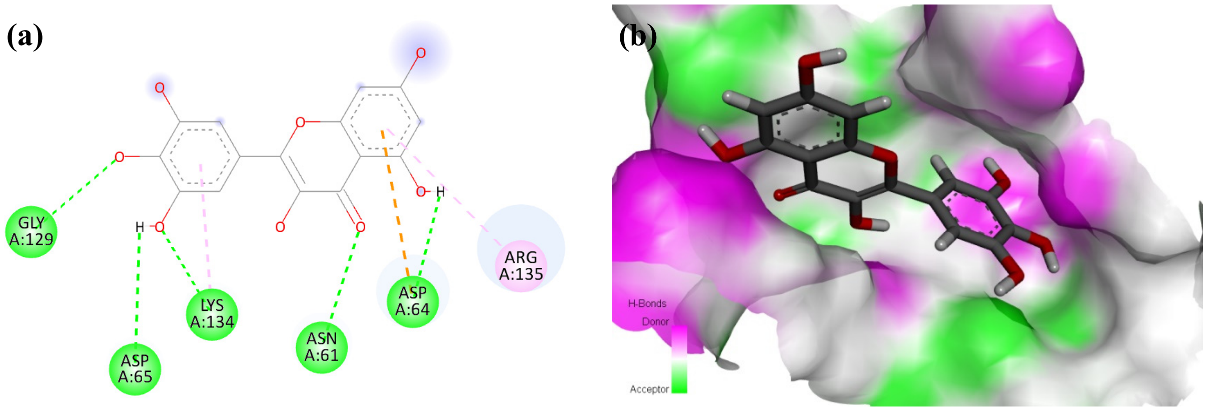

Figure S1. Interaction (a) and binding pattern (b) of myricetin with sex hormone-binding globulin as the receptor.

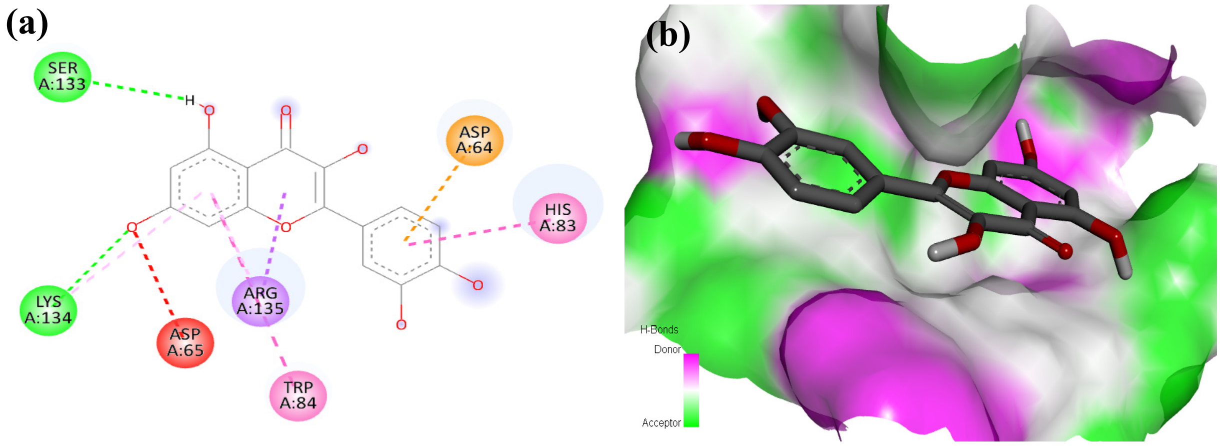

Figure S2. Interactions (a) and binding patterns (b) of quercetin with SHBG as a receptor protein.

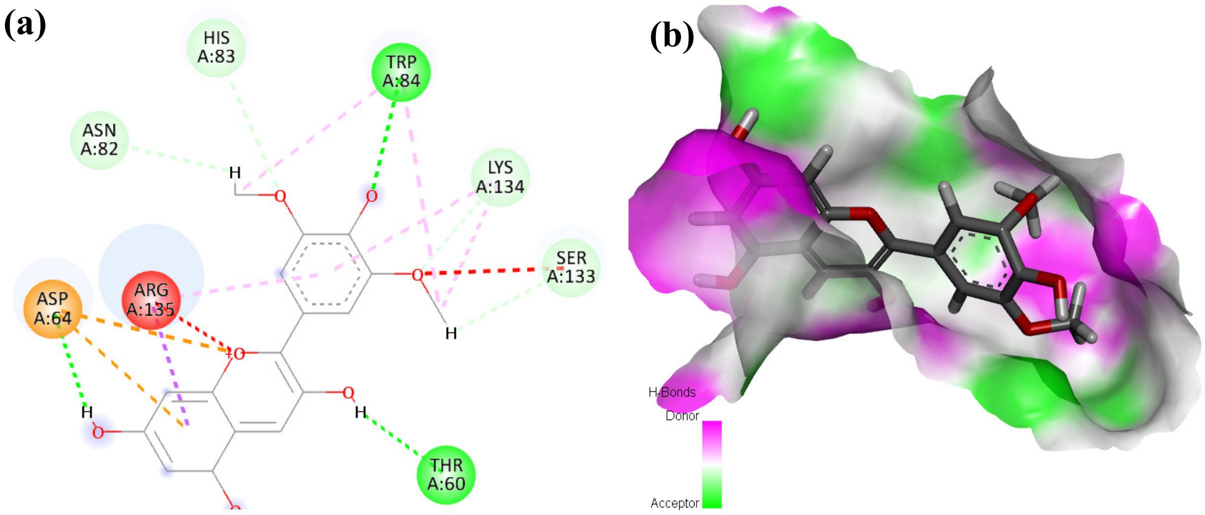

Figure S3. Interactions (a) and binding patterns (b) of malvidin with SHBG as a receptor protein.

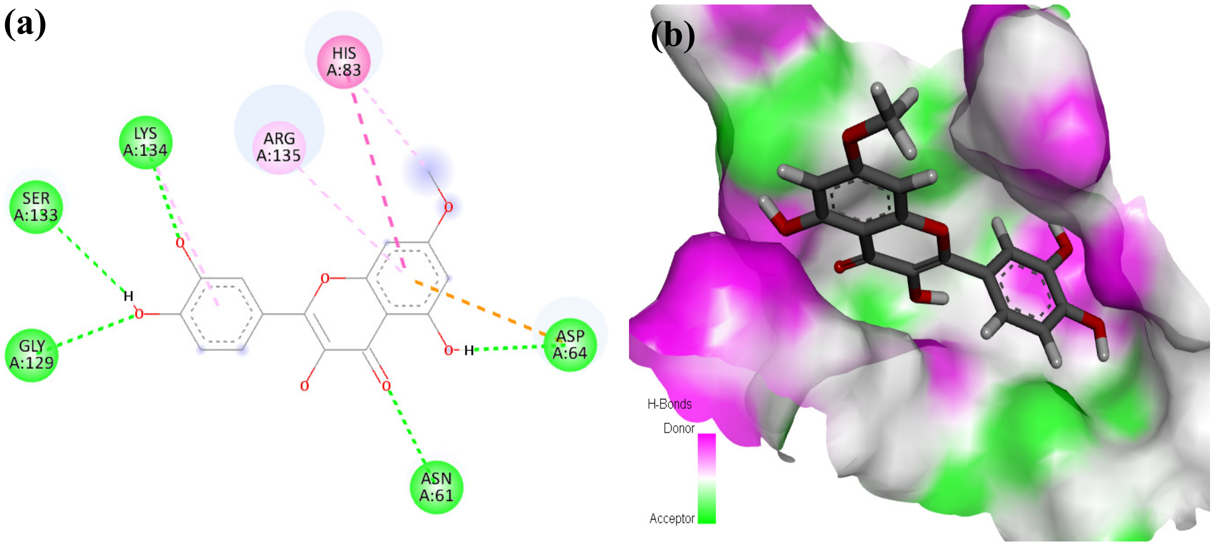

Figure S4. Interactions (a) and binding patterns (b) of rhamnetin with SHBG as a receptor protein.

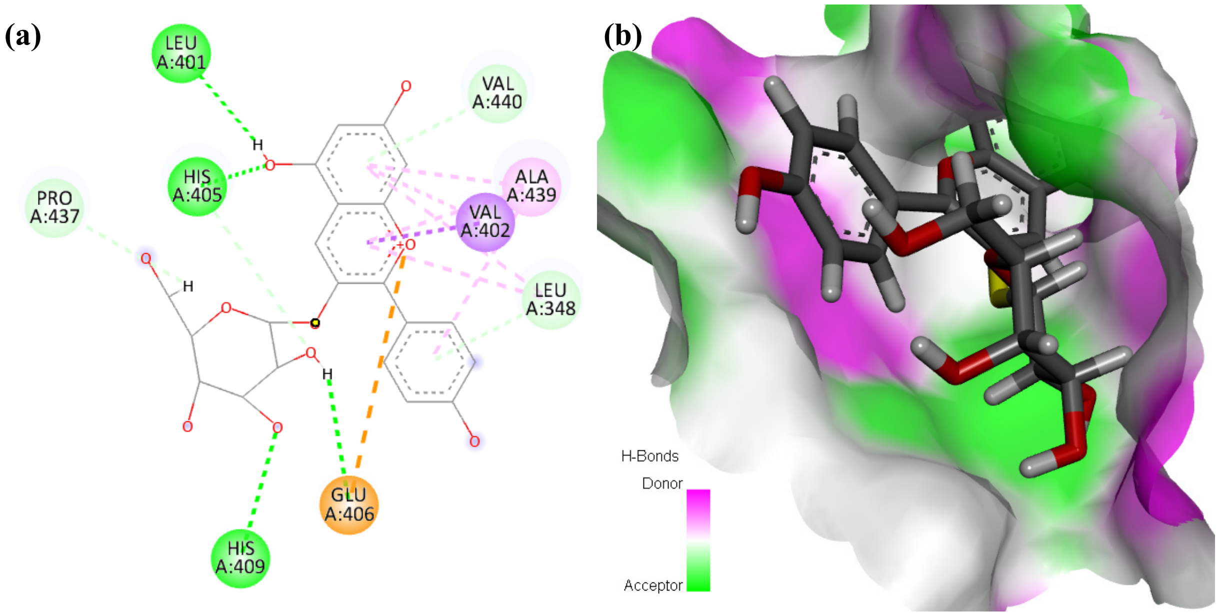

Figure S5. Interaction (a) and binding pattern (b) of callistephin with disintegrin and metalloproteinase 17 (ADAM17) as the receptor.

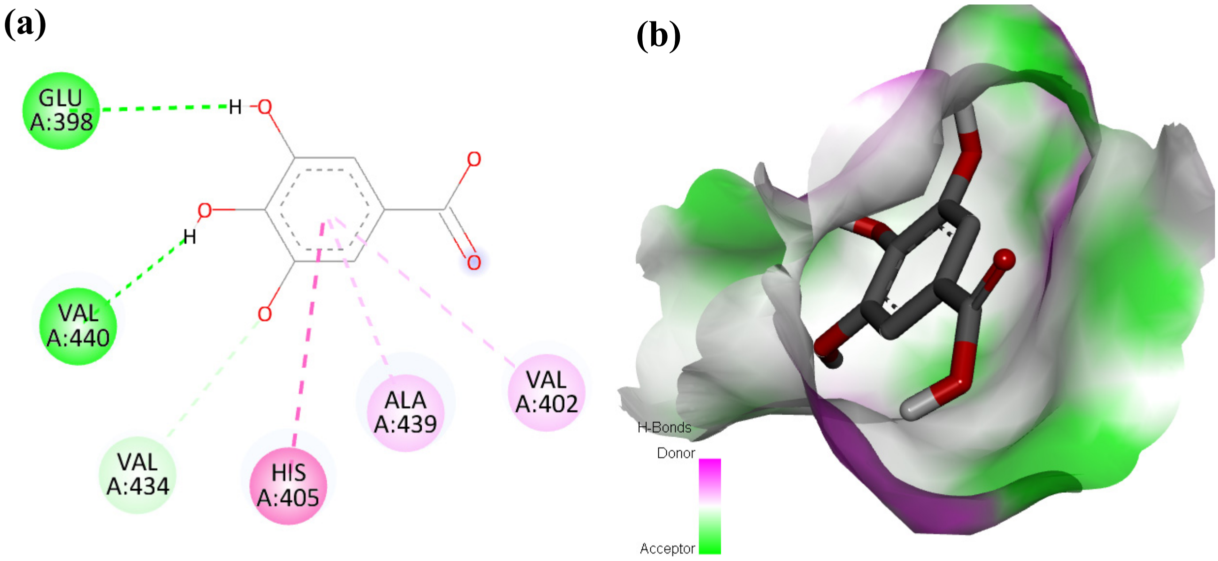

Figure S6. Interactions (a) and binding patterns (b) of gallic acid with ADAM17 as a receptor protein.

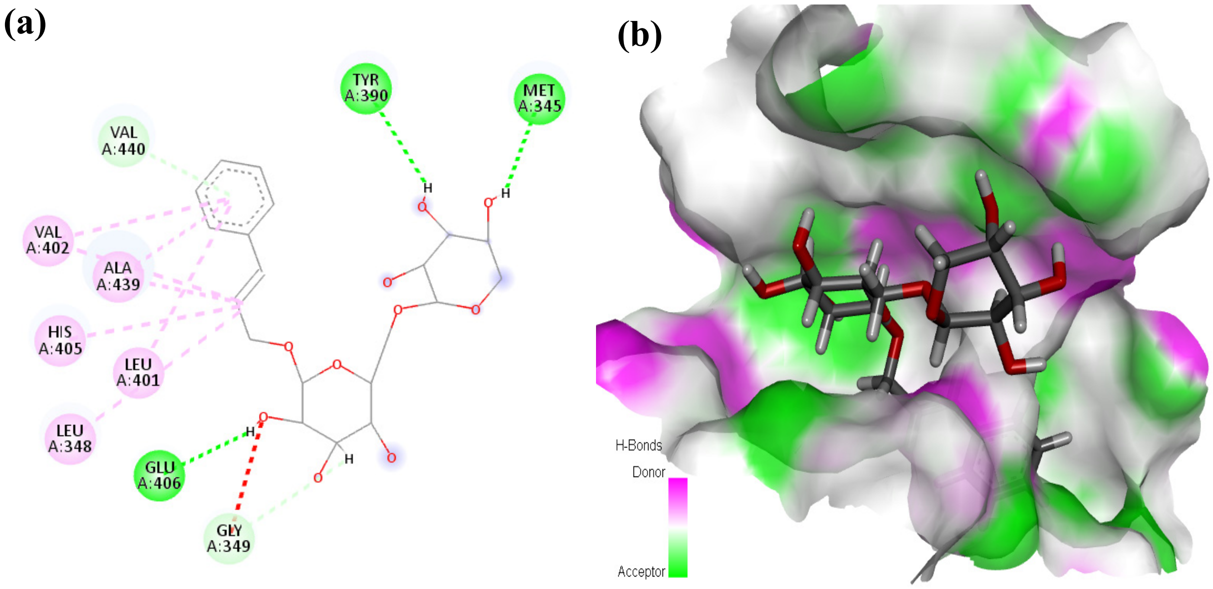

Figure S7. Interactions (a) and binding patterns (b) of rosavin with ADAM17 as a receptor protein.

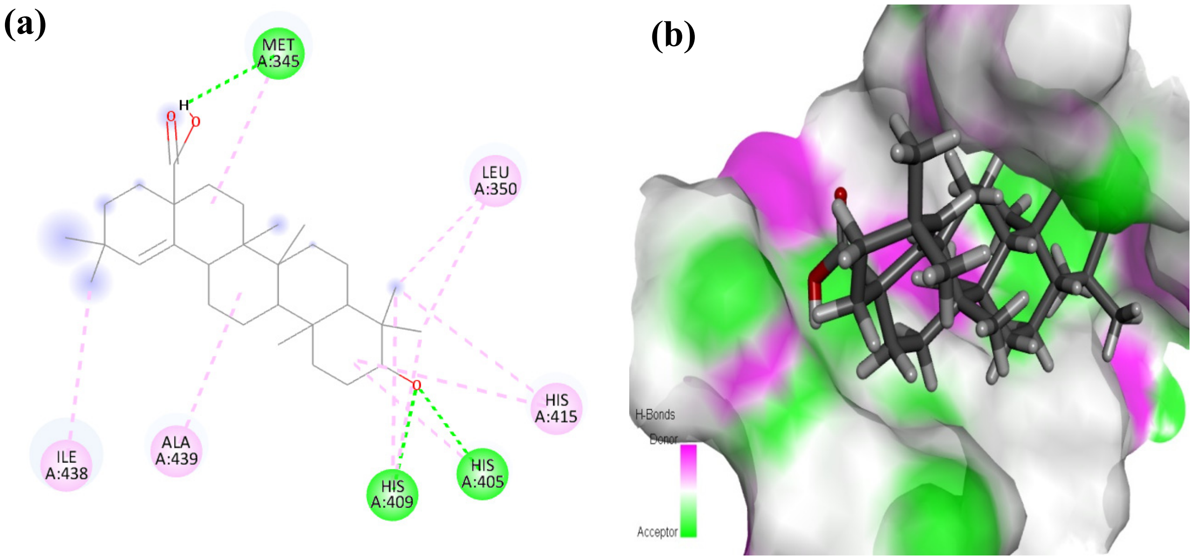

Figure S8. Interactions (a) and binding patterns (b) of moronic acid with ADAM17 as a receptor protein.

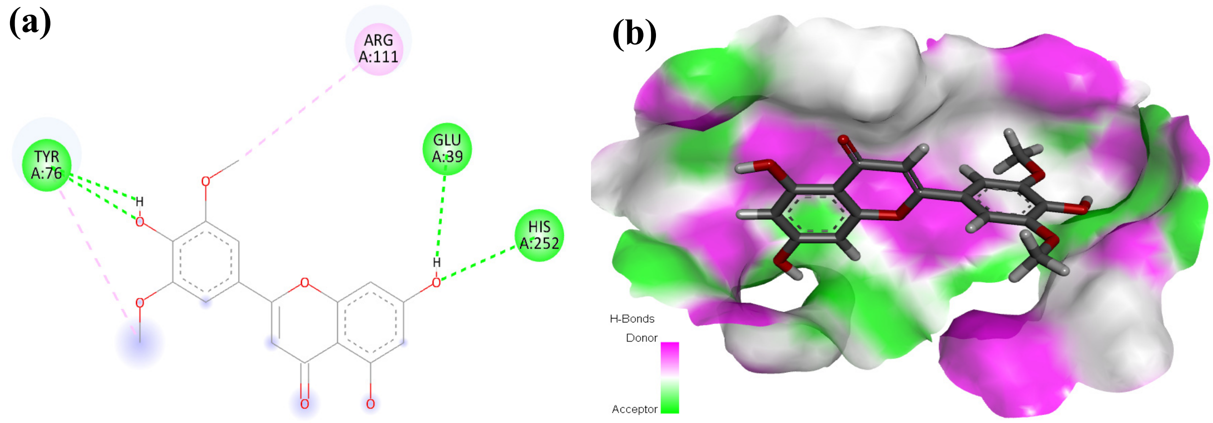

Figure S9. Interactions (a) and binding patterns (b) of tricin with DNaseI as a receptor protein.

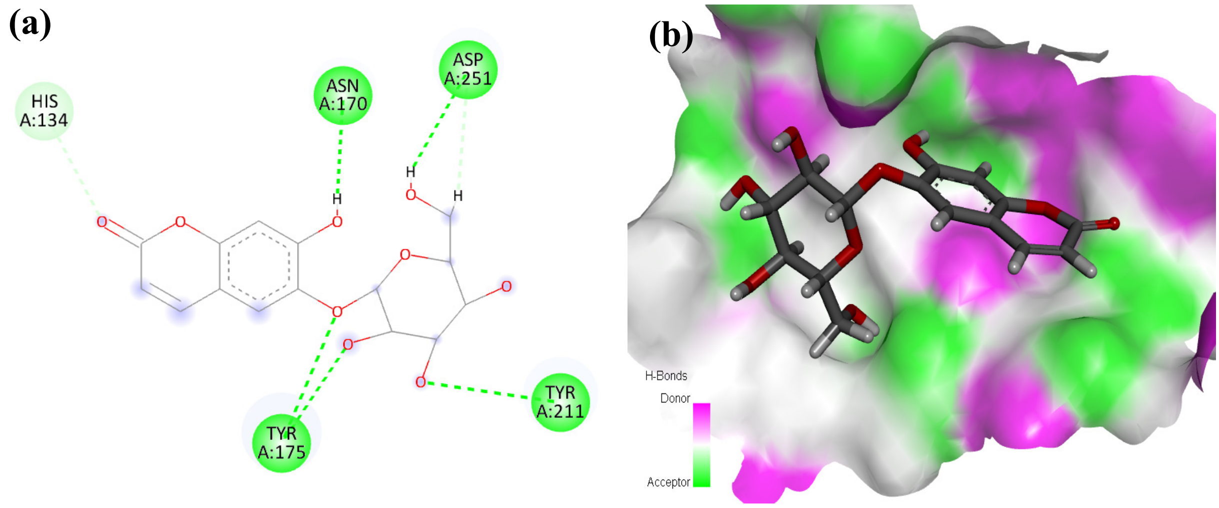

Figure S10. Interactions (a) and binding patterns (b) of esculin with DNaseI as a receptor protein.

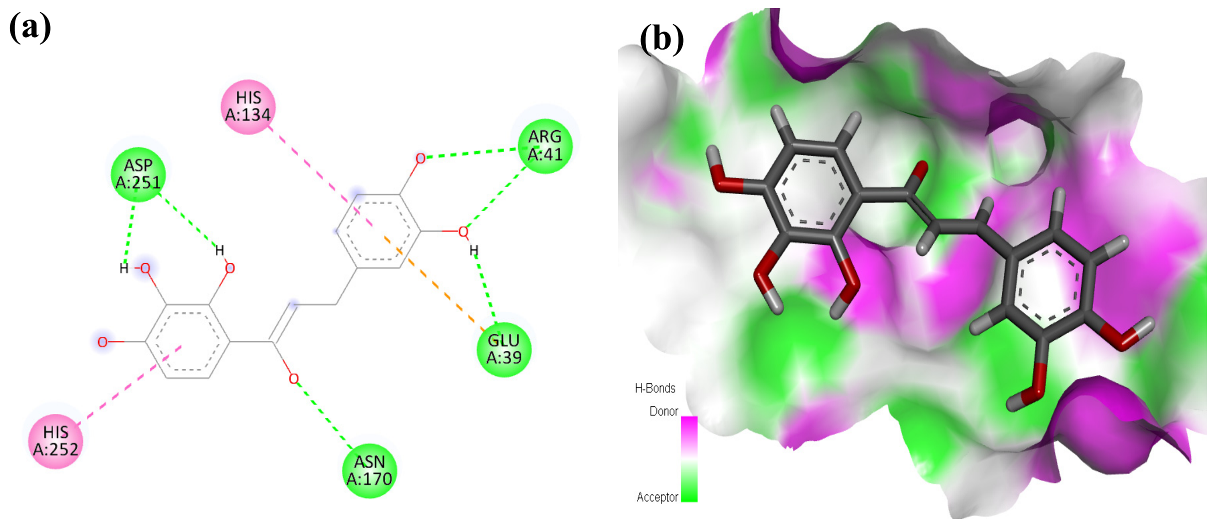

Figure S11. Interactions (a) and binding patterns (b) of okanin with DNaseI as a receptor protein.

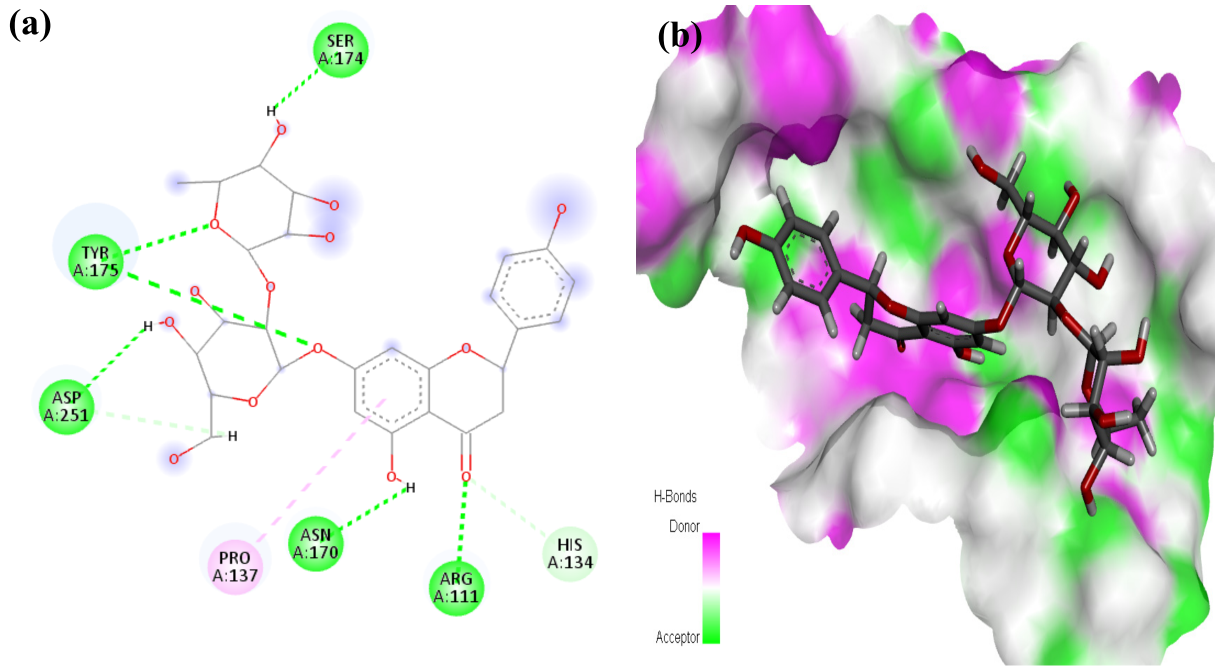

Figure S12. Interactions (a) and binding patterns (b) of naringin with DNaseI as a receptor protein.

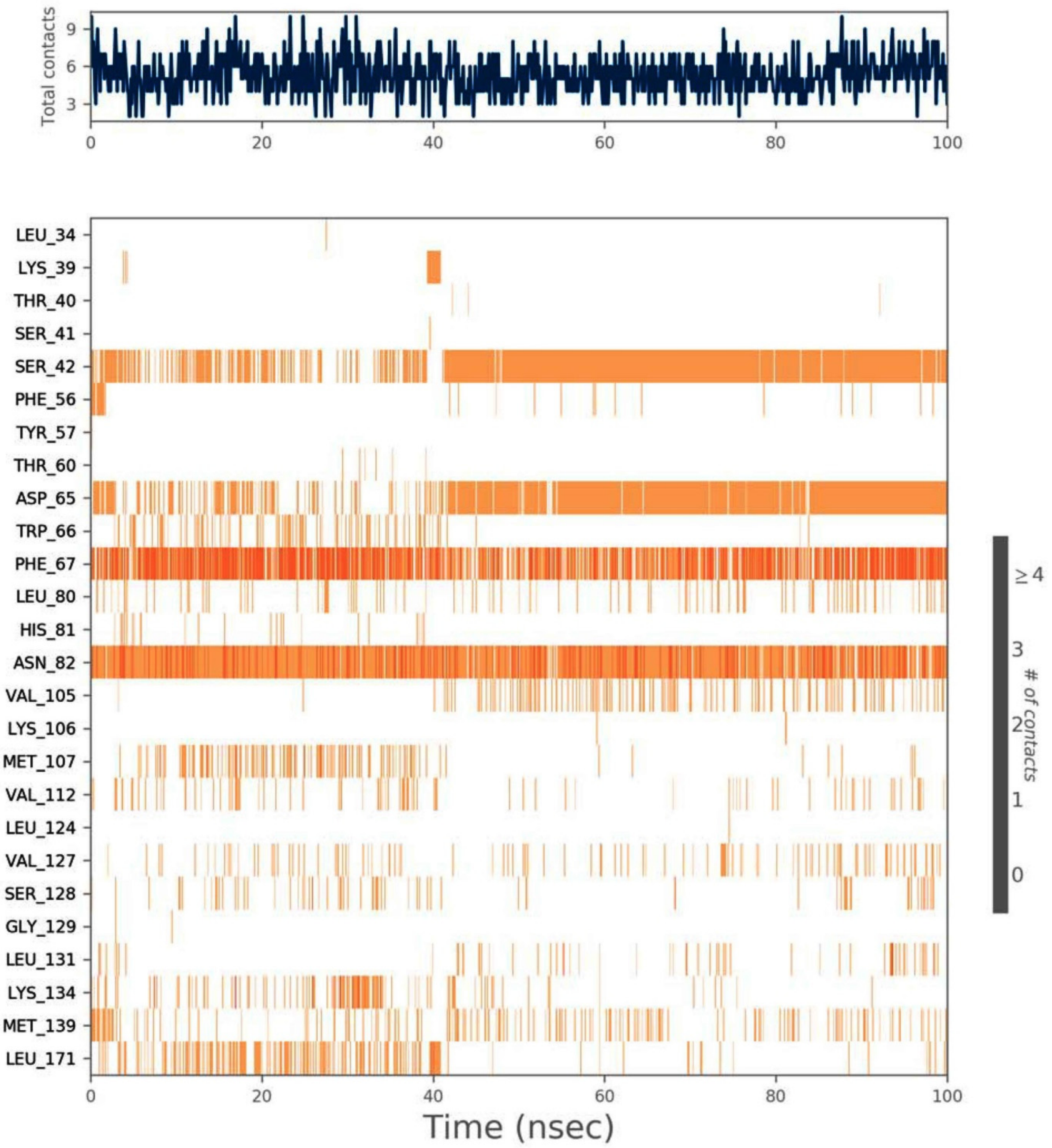

Figure S13. Hydrogen bond interaction stability (consistency) of key residues of SHBG protein with tricin.

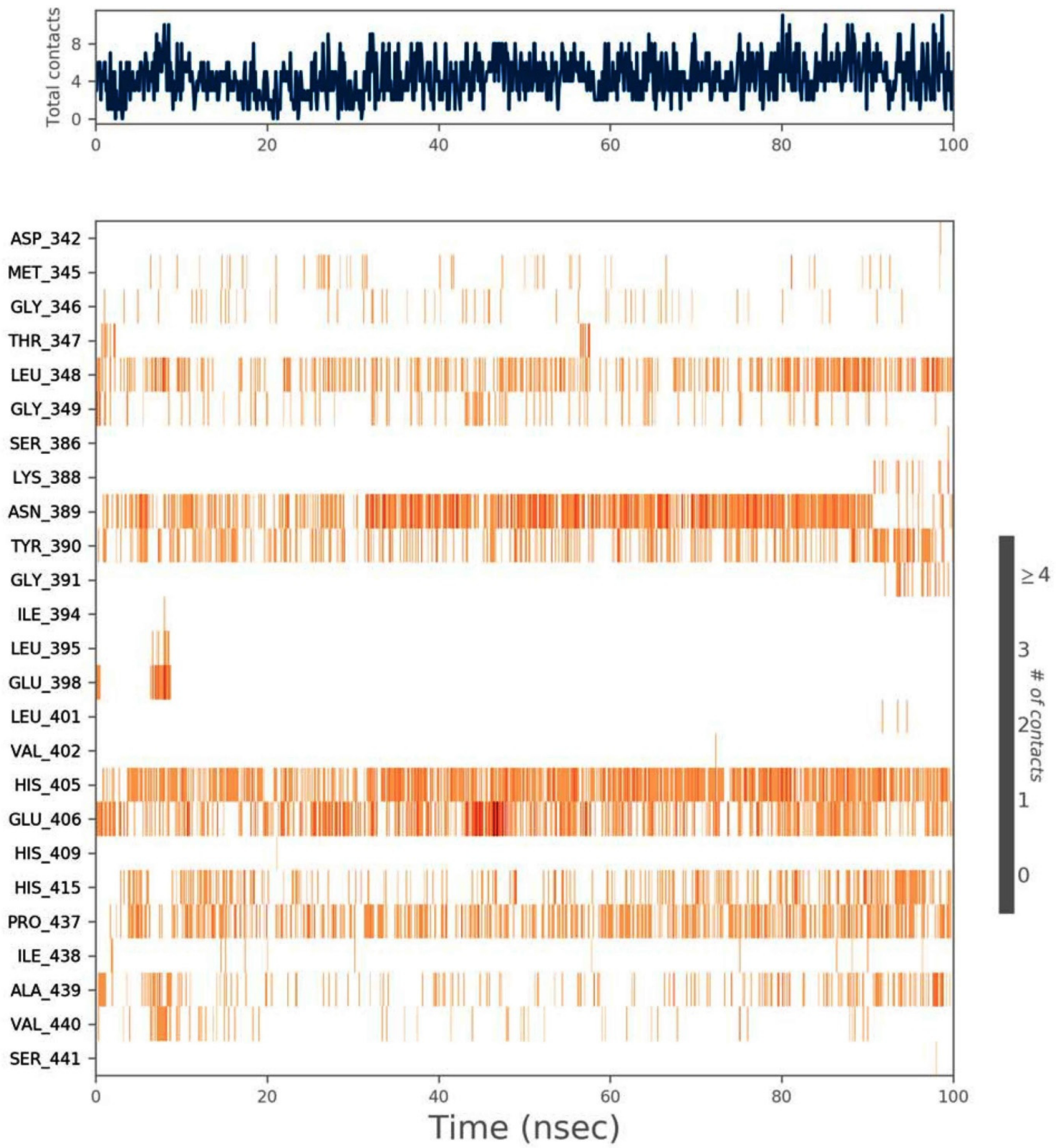

Figure S14. Hydrogen bond interaction stability (consistency) of key residues of the ADAM17 protein with tricin.

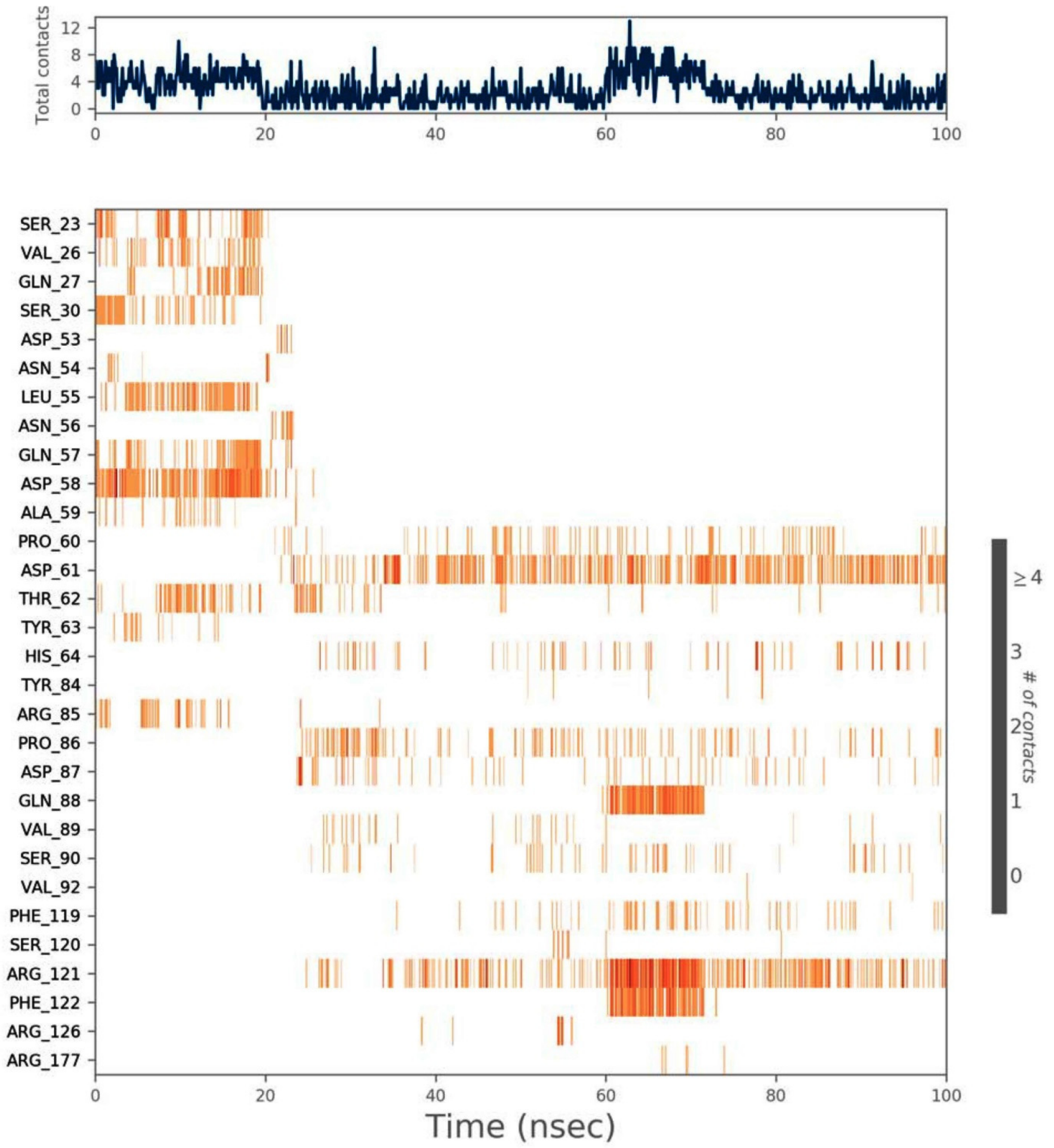

Figure S15. Hydrogen bond interaction stability (consistency) of key residues of the DNaseI protein with tricin.

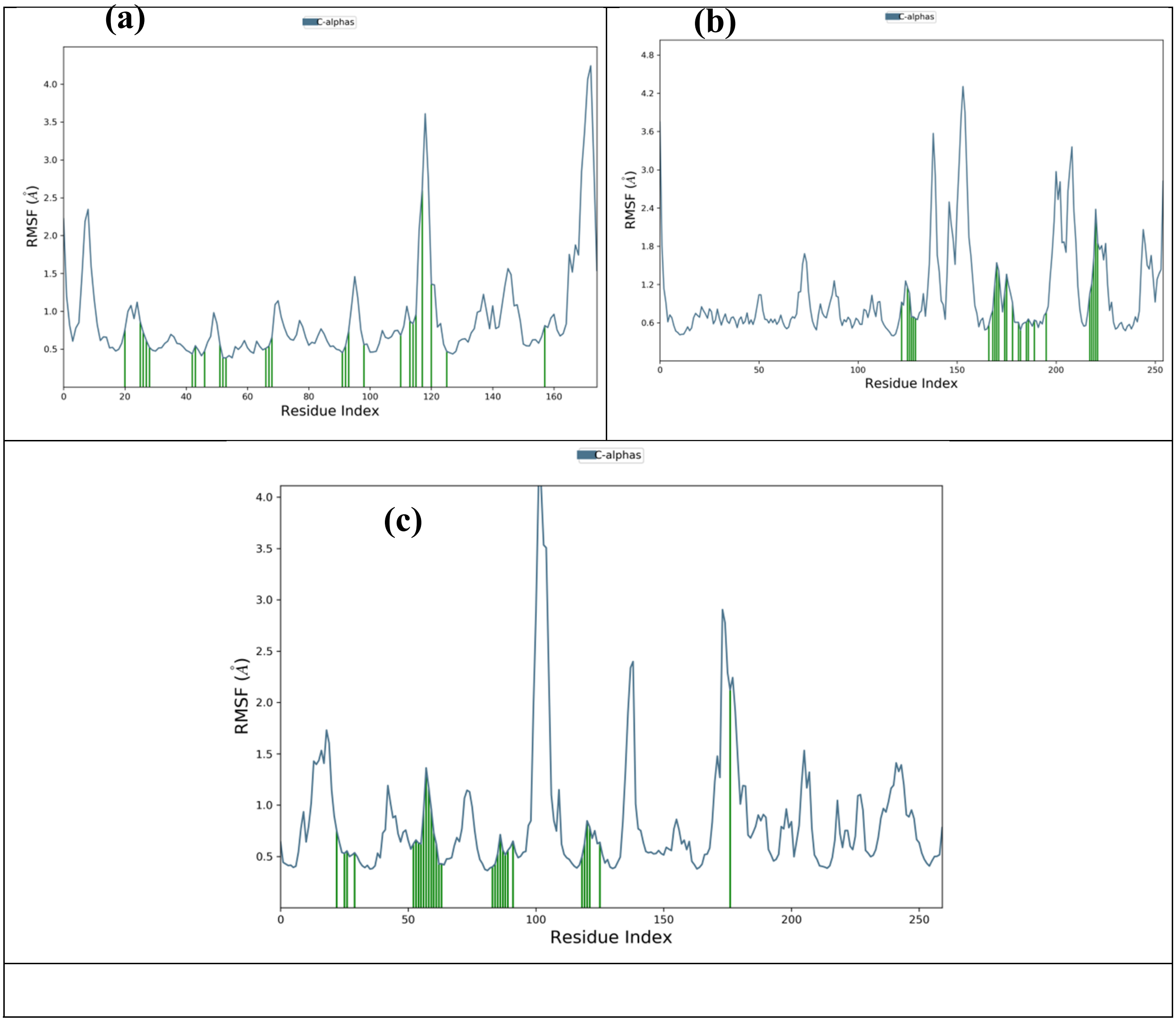

Figure S16. Residue-wise Root Mean Square Fluctuation (RMSF) of protein–ligand complexes during 100 ns. (a) Tricin-SHBG protein; (b) tricin-ADAM17 protein; (c) tricin-DNase I protein. The RMSF of docked protein showed no fluctuation of interacting residues, where green vertical lines represent interacting residues.

Table S1. ADMET screening through pkCSM.

	Myricetin	Malvidin	Rhamnetin	Quercetin	Isorhamnetin	Okanin	Callistephin	kaempferol	Gallic Acid	Rosavin	Esculin	Robinetin	Tricin
GI absorption	Low	High	High	High	High	High	Low	High	High	Low	Low	High	High
BBB	No	No	No	No	No	No	No	No	No	No	No	No	No
Caco-2 Permeability	0.095cm/s	-0.381	-0.361	-0.229	-0.003		0.345	0.032	-0.081	0.269	0.277	-0.563	0.12
PGS	yes	yes	No		Yes	No	Yes	Yes	No	Yes	Yes	Yes	Yes
PGI	No	No	No		No	No	No	No	No	No	No	No	No
CYP2D6 substrate	No	No	No	No	No	No	No	No	No	No	No	No	No
CYP3A4 substrate	No	No	No	No	No	No	No	No	No	No	No	No	No
CYP1A2 inhibitor	Yes	Yes	Yes	Yes	Yes	No	No	Yes	No	No	No	Yes	Yes
CYP2C19 inhibitor	No	No	No	No	No	No	No	No	No	No	No	No	Yes
CYP2C9 inhibitor	No	No	No	No	No	No	No	No	No	No	No	No	No
CYP2D6 inhibitor	No	No	No	No	No	No	No	No	No	No	No	No	No
CYP3A4 inhibitor	No	No	No	No	No	No	No	No	No	No	No	No	No
AMES Toxicity	No	No	No	No	No	No	No	No	No	No	No	No	No
Skin Sensitization	No	No	No	No	No	No	No	No	No	No	No	No	No
Hepatotoxicity	No	No	No	No	No	No	No	No	No	No	No	No	No

Table S2. Medical biochemistry of drugs.

Sr. No.		SA score (<6 easy to synthesize)	Lipinski Rule	Pfizer Rule	GSK Rule	Golden Triangle
1	Myricetin	2.733	Accepted	Accepted	Accepted	Accepted
2	Malvidin	2.944	Accepted	Accepted	Accepted	Accepted
3	Rhamnetin	2.456	Accepted	Accepted	Accepted	Accepted
4	Quercetin	2.545	Accepted	Accepted	Accepted	Accepted
5	Isorhamnetin	2.453	Accepted	Accepted	Accepted	Accepted
7	Okanin	2.392	Accepted	Accepted	Accepted	Accepted
11	Callistephin	4.139	Accepted	Accepted	Rejected	Accepted
12	Kaempferol	2.375	Accepted	Accepted	Accepted	Accepted
13	Gallic Acid	2.095	Accepted	Accepted	Accepted	Accepted
14	Moronic acid	4.653	Accepted	Rejected	Rejected	Rejected
15	Rosavin	4.291	Accepted	Accepted	Rejected	Accepted
16	Esculin	3.732	Accepted	Accepted	Accepted	Accepted
17	Robinetin	2.558	Accepted	Accepted	Accepted	Accepted
18	Tricin	2.430	Accepted	Accepted	Accepted	Accepted
